# Stabilization of 4H hexagonal phase in gold nanoribbons

**DOI:** 10.1038/ncomms8684

**Published:** 2015-07-28

**Authors:** Zhanxi Fan, Michel Bosman, Xiao Huang, Ding Huang, Yi Yu, Khuong P. Ong, Yuriy A. Akimov, Lin Wu, Bing Li, Jumiati Wu, Ying Huang, Qing Liu, Ching Eng Png, Chee Lip Gan, Peidong Yang, Hua Zhang

**Affiliations:** 1 School of Materials Science and Engineering, Nanyang Technological University, 50 Nanyang Avenue, Singapore 639798, Singapore; 2 Institute of Materials Research and Engineering, A*STAR (Agency for Science, Technology and Research), 3 Research Link, Singapore 117602, Singapore; 3 Key Laboratory of Flexible Electronics (KLOFE) & Institute of Advanced Materials (IAM), Jiangsu National Synergistic Innovation Center for Advanced Materials (SICAM), Nanjing Tech University (NanjingTech), 30 South Puzhu Road, Nanjing 211816, China; 4 Institute of High Performance Computing, A*STAR (Agency for Science, Technology and Research), 1 Fusionopolis Way, 16-16 Connexis, Singapore 138632, Singapore; 5 Department of Chemistry, University of California, Berkeley, California 94720, USA; 6 Nanyang Technological University, Temasek Laboratories at NTU, 9th Storey, BorderX Block, Research Techno Plaza 50 Nanyang Drive, Singapore 637553, Singapore

## Abstract

Gold, silver, platinum and palladium typically crystallize with the face-centred cubic structure. Here we report the high-yield solution synthesis of gold nanoribbons in the 4H hexagonal polytype, a previously unreported metastable phase of gold. These gold nanoribbons undergo a phase transition from the original 4H hexagonal to face-centred cubic structure on ligand exchange under ambient conditions. Using monochromated electron energy-loss spectroscopy, the strong infrared plasmon absorption of single 4H gold nanoribbons is observed. Furthermore, the 4H hexagonal phases of silver, palladium and platinum can be readily stabilized through direct epitaxial growth of these metals on the 4H gold nanoribbon surface. Our findings may open up new strategies for the crystal phase-controlled synthesis of advanced noble metal nanomaterials.

## Abstract (web summary)

Noble metals typically crystallize with the face-centered cubic structure. Here, the authors report the synthesis of gold nanoribbons in the 4H hexagonal polytype, a previously unreported, metastable phase of gold, and use it to stabilize 4H hexagonal phases of silver, palladium and platinum.

## Graphical abstract

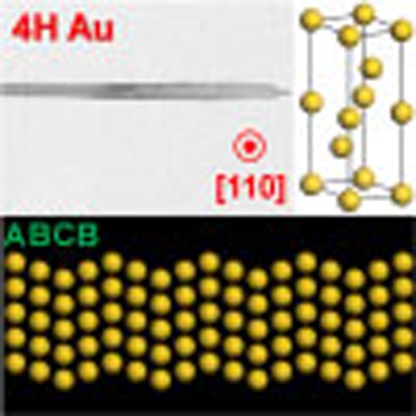

Control over crystal structures is one of the key challenges in the synthesis of functional inorganic nanostructures^1^,^2^,^3^. Polymorphism is commonly observed in II–VI, III–V, IV and transition metal oxide semiconductor nanocrystals^4^,^5^,^6^,^7^,^8^. The crystal structure of semiconductor nanocrystals is often dictated by experimental factors such as the reaction temperature^5^ and pressure^4^,^8^, solvent system^9^, the interaction between crystal nuclei and capping agents^10^ and crystal size^5^,^7^. Although the polymorphs of a particular semiconductor show the same chemical composition, their physical properties may significantly differ from each other^3^,^6^. For example, the band gap of wurtzite SiC is 3.33 eV, which is much larger than that of the zinc-blende counterpart (2.39 eV; ref. 6). Different from semiconductors, the structural tuning of noble metals still remains challenging, especially for nanostructures synthesized using wet-chemical methods. As known, the functional properties of metal nanostructures are closely related to their crystal structures^11^,^12^. For instance, face-centred tetragonal FePt nanoparticles (NPs) demonstrate a superior catalytic activity and durability for oxygen reduction reaction compared with the common face-centred cubic (*fcc*) counterparts^11^. Recently, it was reported that hexagonal close-packed (*hcp*) Ru NPs with size of less than 3 nm are much more catalytically active towards CO oxidation compared with the *fcc* Ru NPs with the similar size^12^. Therefore, it is of paramount importance to achieve the phase-controlled synthesis of metal nanostructures.

Gold (Au) nanostructures have received extensive attention because of their attractive properties for many applications including biosensing, catalysis, surface-enhanced Raman scattering and biomedicine^13^,^14^,^15^. Decades ago, well-dispersed Au NPs were obtained by reduction of HAuCl_4_ with sodium citrate^16^. After that, various methods, such as the porous aluminium anodic oxide-directed hard-templating approach^17^, surfactant-driven soft-templating synthesis^18^ and seed-mediated growth^19^, have been developed to synthesize various anisotropic Au nanostructures, for example, rods^18^,^19^, plates^20^,^21^, wires^17^,^22^,^23^,^24^, ribbons^25^ and polyhedra^26^,^27^. However, almost all the Au nanostructures reported so far crystallize in the common *fcc* structure. In contrast, the non-*fcc* polytypes of Ag, such as hexagonal 2H and 4H with a characteristic stacking sequence of ‘AB' and ‘ABCB' along their close-packed directions, respectively, have been obtained with different synthetic methods such as high-pressure direct current magnetron sputtering^28^ and porous aluminium anodic oxide-assisted electrochemical deposition^29^,^30^,^31^,^32^. It was found that 4H Ag showed 130 times greater in-plane resistivity and much stronger surface plasmon absorption compared with the common *fcc* Ag^32^. Different with Ag, the 4H polytype of Au has not been discovered so far. Very recently, for the first time, our group reported the wet-chemical synthesis of pure *hcp* (2H type) Au square sheets^33^, which inspires the exploration of new polymorphs of Au and study of their structural modulation.

Here we report the high-yield colloidal synthesis of hexagonal 4H Au nanoribbon (NRB), another new metastable phase of Au. We observe that the 4H structure of Au NRBs can be transformed to the *fcc* structure on ligand exchange under ambient conditions. In addition, the existence of well-developed surface plasmon resonance (SPR) on a single 4H Au NRB is demonstrated using monochromated electron energy-loss spectroscopy (EELS). It is further demonstrated that 4H hexagonal phase of Ag, Pd and Pt can be readily stabilized through direct epitaxial growth of these metals on our 4H Au NRB surface.

## Results

### Synthesis and characterization of 4H Au NRBs

**Figure 1:**
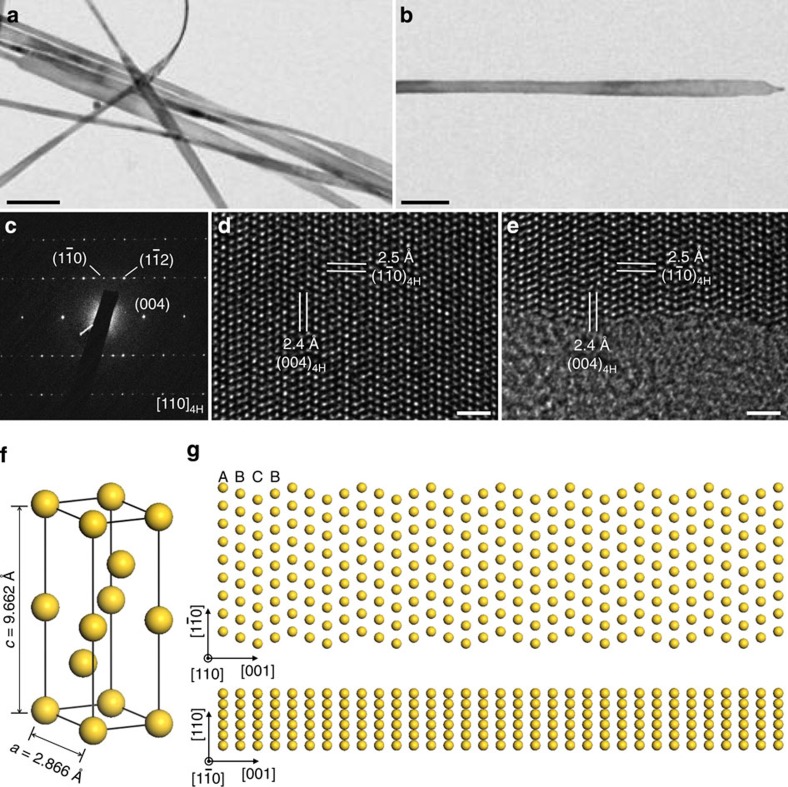
TEM analysis and crystal models of 4H Au NRBs. (**a**) A typical TEM image of 4H Au NRBs (scale bar, 100 nm). (**b**,**c**) TEM image (scale bar, 100 nm) and the corresponding SAED pattern taken along the [110]_4H_ zone axis of a typical Au NRB. (**d**,**e**) Aberration-corrected HRTEM images taken in the centre and at the edge of an Au NRB, respectively (scale bars, 1 nm). (**f**) Schematic illustration of a unit cell of 4H Au. The simulated unit cell parameters are *a*=2.866 Å and *c*=9.662 Å. (**g**) Crystallographic models illustrating the top view (top panel) and side view (bottom panel) of a typical 4H Au NRB. The close-packed planes along the [001]_4H_ direction show a characteristic stacking sequence of ‘ABCB'.

The Au NRBs were prepared by heating the mixture of HAuCl_4_, oleylamine, hexane and 1,2-dichloropropane in a closed glass vial at 58 °C for 16 h (see Methods for details). The obtained Au NRBs with length of 0.5–6.0 μm and width of 15.0–61.0 nm are confirmed using the transmission electron microscope (TEM) images (Fig. 1a,b and Supplementary Fig. 1). The thickness of the Au NRBs is estimated to be 2.0–6.0 nm from TEM images of their folded edges (Supplementary Fig. 2), which is further confirmed using atomic force microscopy (AFM) imaging (Supplementary Fig. 3). Scanning TEM-energy dispersive X-ray spectrum (STEM-EDS) reveals that the chemical composition of as-prepared NRBs is pure Au (Supplementary Fig. 4). The surface of Au NRBs is capped by the oleylamine molecules, as evidenced using the X-ray photoelectron spectroscopy analysis (Supplementary Fig. 5). Note that the use of 1,2-dichloropropane is essential for the synthesis of Au NRBs. Without the addition of 1,2-dichloropropane, only twinned *fcc* Au NPs and very small amount of ultrathin Au nanowires (NWs) were obtained (Supplementary Fig. 6).

The as-prepared Au NRB crystallizes in an unprecedented 4H hexagonal polytype (space group P6_3_/mmc), as corroborated using the selected area electron diffraction (SAED) pattern taken along the [110]_4H_ zone axis (Fig. 1c), which exhibits diffraction spots of the (004)_4H_, 
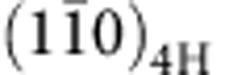
 and 
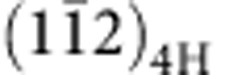
 planes. The SAED pattern and corresponding TEM image of an Au NRB reveal that it grows along the [001]_4H_ direction (Fig. 1b,c). To further confirm the 4H structure of Au NRBs, the X-ray diffraction pattern of the Au nanostructures, deposited on a glass substrate, was also collected. The obtained X-ray diffraction pattern clearly showed four prominent peaks located at 36.2°, 37.4°, 40.9° and 79.7°, which can be assigned to the (100)_4H_, (101)_4H_, (102)_4H_ and (202)_4H_ planes of 4H Au, respectively (Supplementary Fig. 7). Simulated unit cell parameters of 4H Au are *a*=2.866 Å and *c*=9.662 Å (Fig. 1f). The other peaks located at 38.2°, 44.3° and 77.6° are from the coexisting *fcc* Au NPs, that is, the by-products obtained during the synthesis of Au NRBs (Supplementary Fig. 1). Note that the peak at 64.8° is attributed to both (110)_4H_ and (220)_f_ planes as their lattice spacing is equal. The aberration-corrected high-resolution TEM (HRTEM) images show that the 4H structure extends from the centre to the edge region of a typical Au NRB (Fig. 1d,e), which has been further identified with the simulated HRTEM image (Supplementary Fig. 8). On the basis of these observations, crystal structure models (top and side views) of an Au NRB are proposed, illustrating the characteristic stacking sequence of ‘ABCB' along the close-packed [001]_4H_ direction (Fig. 1g).

### Formation mechanism of 4H Au NRBs

In order to understand the formation mechanism of 4H Au NRBs, time-dependent experiments were conducted at intervals of 4 h, and the intermediate products were examined using TEM (Supplementary Figs 9 and 10). At the reaction time of 4 h, ultrathin Au NWs (1.4–2.0 nm in diameter) were formed (Supplementary Fig. 9a), which then grew into ribbon-like structures with width increased to 2.8–5.2 nm at the reaction time of 8 h (Supplementary Fig. 9b). With further increase in reaction time, the width of the Au NRBs continued to increase to 8.0–20.0 nm (Supplementary Fig. 9c) and 15.0–61.0 nm (Supplementary Fig. 9d) at 12 and 16 h, respectively. Earlier studies have suggested that the formation of ultrathin Au NWs may be driven by the soft-templating effect of one-dimensional polymer structures, which were assembled from (oleylamine)AuCl complex molecules via the aurophilic interaction^22^,^24^,^34^. In the present work, the evolution of the ultrathin Au NWs into Au NRBs is also likely assisted by the polymeric template. In addition to the morphological change, that is, from the ultrathin NWs to NRBs, the transformation of the crystal structure is also observed. The ultrathin Au NWs as indicated using HRTEM image (Supplementary Fig. 10a) contain hexagonal 2H domains together with random stacking faults, which is also confirmed in our previous study^35^. As the Au NWs grew and started to adopt the ribbon-like morphology with width of 2.8–5.2 nm (Supplementary Fig. 9b), 4H domains started to appear (Supplementary Fig. 10b). When the width of Au NRBs further increased to 8.0–20.0 nm (Supplementary Fig. 9c), 4H Au NRBs were successfully obtained (Supplementary Fig. 10c).

Previously, the 2H (with a stacking sequence of AB) to 4H phase (with a stacking sequence of ABCB) transformation has been observed in materials such as TiCr_2_ via shear-induced formation of regular stacking faults^36^. In addition, the 2H-to-4H transition induced by thermal energy was also observed in the transformation of PbI_2_, whose 4H phase is more stable than the 2H phase at the transition temperature^37^. Similarly, the transformation of 2H ultrathin Au NWs to 4H Au NRBs could also be driven by their energy difference, since previous theoretical calculations predicted that 4H Au is relatively more stable than 2H Au because of its larger cohesive energy per atom^30^.

### Ligand exchange-induced phase transformation of 4H Au NRBs

**Figure 2:**
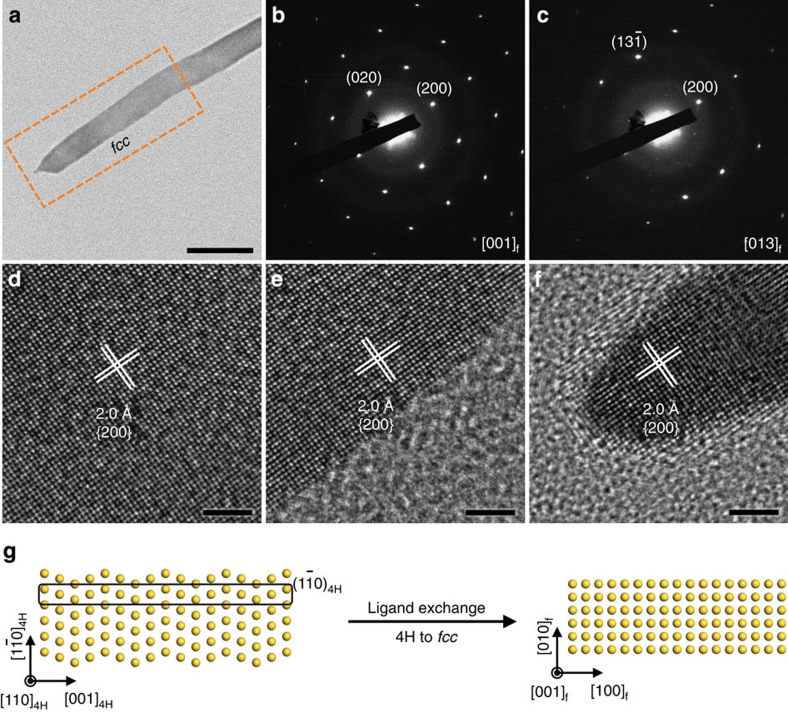
The ligand exchange-induced phase transformation of Au NRBs. (**a**) A typical TEM image of an Au NRB after the ligand exchange (scale bar, 50 nm). (**b**) The corresponding SAED pattern of the region inside the dashed rectangle in **a**, showing the *fcc* structure oriented along the [001]_f_ zone axis. (**c**) SAED pattern of the [013]_f_ zone axis was taken by tilting the Au NRB around the [200]_f_ zone axis by 18.2° with respect to the [001]_f_ zone axis. (**d**–**f**) HRTEM images taken from the centre, edge and end of the marked region in **a**, respectively (scale bars, 2 nm). (**g**) Schematic illustration of the ligand-induced phase change of Au NRB.

The 4H structure of Au NRBs is metastable, and can be transformed to the *fcc* structure on the exchange of surface-capped amine molecules with thiol molecules under ambient conditions (Fig. 2). Typically, the ligand exchange was conducted by vortex-mixing the Au NRB solution and a fresh 1-dodecanethiol solution for 5 min. The thiol-treated Au NRBs were analysed using STEM-EDS, which indicated the presence of sulfur in the obtained product and thus confirmed the occurrence of the ligand exchange (Supplementary Fig. 11). The structure of thiol-treated Au NRBs is found to be *fcc*, which is proven by the SAED pattern taken on the highlighted area in Fig. 2a, showing the square lattice pattern along the [001]_f_ zone axis (Fig. 2b). The (001)_f_-oriented *fcc* structure is further verified using the SAED pattern taken along the [013]_f_ zone axis, obtained by tilting the Au NRB around the [200]_f_ axis by 18.2°, which matches well with the theoretical angle of 18.4° between [001]_f_ and [013]_f_ zone axes (Fig. 2c). HRTEM images further reveal the (001)_f_-oriented *fcc* structure with lattice spacing of 2.0 Å for {200} planes (Fig. 2d–f). Figure 2g and Supplementary Fig. 12 schematically demonstrate the thiol-induced phase transformation of Au NRB from the original (110)_4H_-oriented 4H to the (001)_f_-oriented *fcc* structure. Different from the commonly observed *hcp*-to-*fcc* phase transformation in metals that proceeds by motion of partial dislocations on the close-packed planes and results in the formation of stacking faults/twins^38^, the phase transition of Au NRBs might arise from the flattening of the 
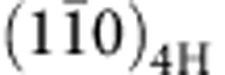
 planes (indicated by the rectangle in the left image of Fig. 2g), which is similar to the wurtzite-to-rock salt transition occurring in some semiconductor nanocrystals under high pressure, such as GaN and CdSe (refs 4, 39). Previous studies suggested that thiols and other sulfur-containing molecules can induce surface reconstruction of metals, especially favouring the generation of overlayers with high coordination numbers, such as *fcc*(100) that contains a square hollow site^40^. In addition, spherical Pt NPs were observed to transform to Pt nanocubes enclosed by {100}_f_ facets in the presence of H_2_S at 500 °C (ref. 41). Therefore, the ligand-induced phase change of Au NRBs under ambient conditions is likely driven by the particularly strong and unique interaction between Au and S (refs 40, 42).

### Monochromated EELS study of an individual 4H Au NRB

**Figure 3:**
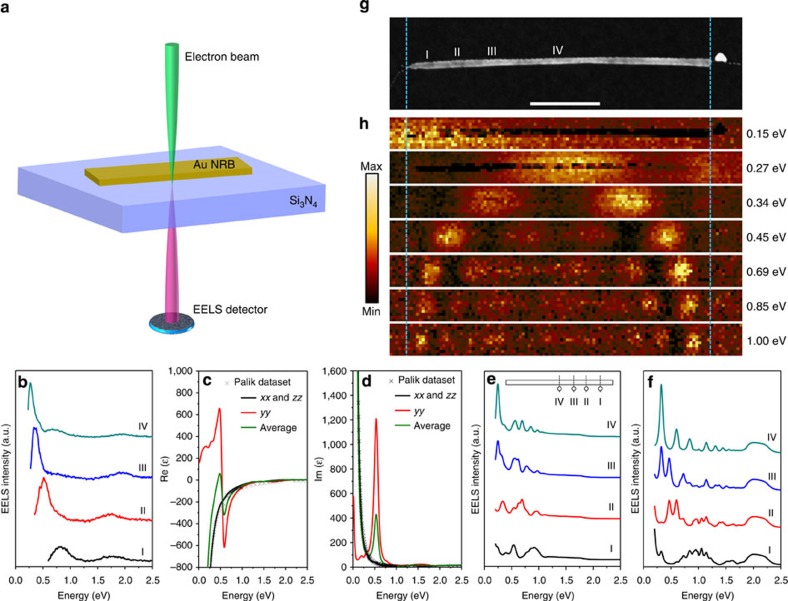
SPR analysis of single 4H Au NRB. (**a**) Schematic illustration of monochromated EELS measurement on a single Au NRB. The focused electron beam is located next to the Au NRB to excite and measure its SPR. (**b**) EELS spectra acquired from an individual Au NRB excited at the positions indicated in the STEM image shown in **g**. (**c**,**d**) DFT-calculated dielectric function of 4H Au thin film with the limitation of 4 nm in its *y* direction. (**e**) Calculated EELS spectra of a 4H Au NRB based on dielectric function in **c**,**d** using the FEM with an electron beam. Inset: the theoretical model of an individual Au NRB, in which the excitation positions are marked. (**f**) Calculated EELS spectra of an *fcc* Au NRB with dielectric function taken from Palik at the same excitation positions as **e**. (**g**,**h**) HAADF-STEM image of single Au NRB (scale bar, 200 nm) and its corresponding EELS maps at different energy loss.

Single 4H Au NRB was further examined using the monochromated EELS in TEM (Fig. 3a, see Methods for details). The STEM-EELS measurement was performed by raster-scanning a focused electron beam with diameter of 1–2 nm in a rectangular region that included a single 4H Au NRB. The interaction of electron beam with the conduction electrons in Au NRB generated the localized SPR, which provided a fingerprint of the optoelectronic property of Au NRB. Figure 3b shows the EELS spectra of a single Au NRB collected from different positions (that is, I, II, III and IV), a few nanometres next to the Au NRB, as indicated in Fig. 3g. Two distinct SPR peaks were observed in all EELS spectra, that is, 0.82 and 1.75 eV at position ‘I', 0.52 and 1.72 eV at position ‘II', 0.36 and 1.93 eV at position ‘III' and 0.27 and 1.94 eV at position ‘IV'. The SPR peaks of the Au NRB at different excitation positions that appeared at low and high energy can be attributed to the eigenmodes longitudinally and transversely polarized with respect to the NRB geometry, respectively^43^. There is a remarkable red shift for the dominant SPR peak from excitation position ‘I' to position ‘IV'. As there is not any optical data available for 4H Au NRBs, density functional theory (DFT) calculations were used to determine the dielectric function of a 4H Au thin film (Fig. 3c,d, see Methods for details). In DFT modelling, we define the 
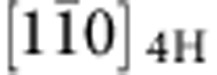
 to be *x* direction, [001]_4H_ to be *y* direction and [110]_4H_ to be *z* direction (Fig. 1c,f). The 4H Au thin film is assumed to have 4 nm in its *y* direction and stack indefinitely in its *x* and *z* directions. The dielectric function of 4H Au thin film and that of *fcc* Au (obtained from literature, ref. 44) show different features in the perpendicular direction to the thin film (that is, the *yy* direction). Such difference comes from surface atoms with the formation of plasma frequency at low frequency. The contribution of surface atoms to the dielectric function is smaller with increasing of thin film thickness^45^. A red shift for the primary SPR peak from excitation position ‘I' to position ‘IV', which is consistent with the experimental result (Fig. 3b), was also observed in our finite-element-method (FEM) simulation of plasmon excitation with an electron beam (Fig. 3e, see Methods for details). Note that the Palik's frequency-dependent dielectric function, obtained from *fcc* Au (ref. 44), was also used in our FEM simulations to benchmark the difference of optical response between 4H Au and *fcc* Au (Fig. 3f). As compared with *fcc* Au (Fig. 3f), the optical response of 4H Au thin film shows a smaller number of SPR peaks in the range of 0–2.5 eV and red shift for all SPR peaks from position ‘I' to position ‘IV' (Fig. 3e). The experimental EELS maps further reveal that low-order harmonic resonances were formed using low-energy SPR eigenmodes and the harmonic order increased with the SPR energy (Fig. 3h). The critical insight gained here is that the simulations with DFT-calculated optical constants of 4H Au followed more closely to the trend of experiments. Since the optical responses of 4H Au and *fcc* Au are distinctive in terms of position and magnitude of SPR peaks, it is reasonable to conclude that the structure tuning of Au can significantly alter its optical properties.

### Synthesis of 4H hexagonal structures of other noble metals

**Figure 4:**
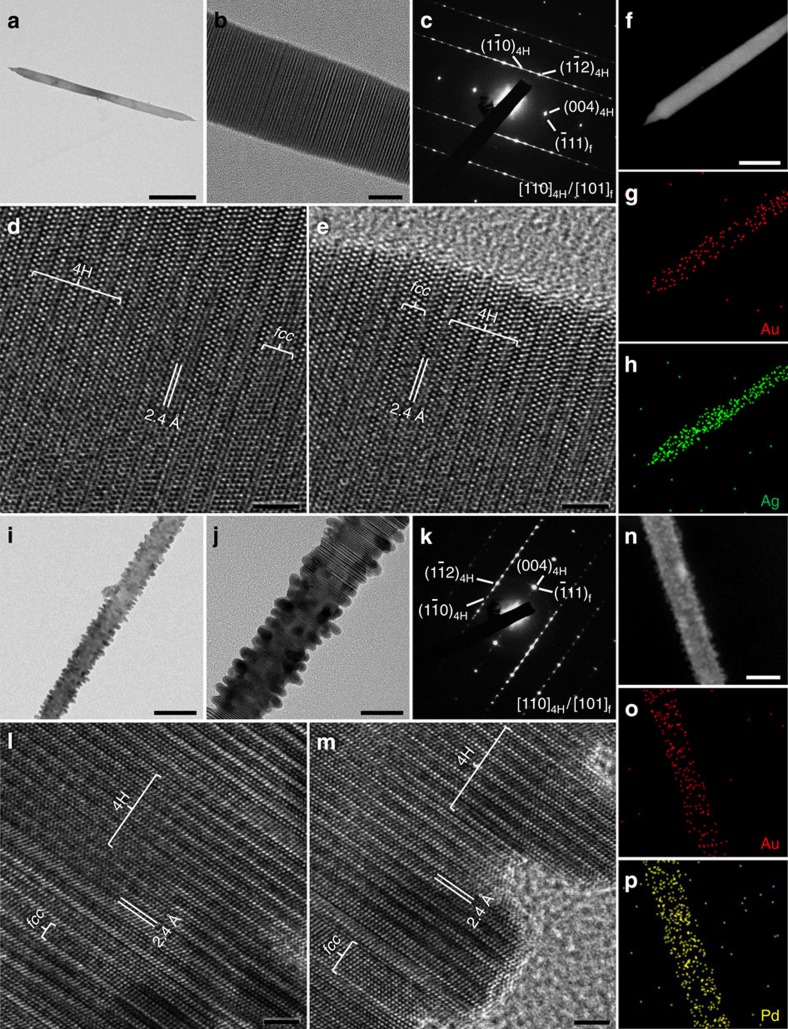
TEM analysis of bimetallic 4H/*fcc* Au@Ag and Au@Pd NRBs. (**a**) Bright-field TEM image of a typical Au@Ag NRB (scale bar, 200 nm). (**b**) Magnified TEM image of an Au@Ag NRB (scale bar, 10 nm). (**c**) A typical SAED pattern of an Au@Ag NRB taken along the [110]_4H_/[101]_f_ zone axes. (**d**,**e**) HRTEM images of an Au@Ag NRB taken in the centre and at the edge, respectively (scale bars, 2 nm). (**f**) HAADF-STEM image (scale bar, 100 nm) and (**g**,**h**) the corresponding STEM-EDS elemental mappings of a typical Au@Ag NRB. (**i**) A typical bright-field TEM image of an Au@Pd NRB (scale bar, 50 nm). (**j**) Magnified TEM image of a typical Au@Pd NRB (scale bar, 20 nm). (**k**) SAED pattern of a typical Au@Pd NRB collected along the [110]_4H_/[101]_f_ zone axes. (**l**,**m**) HRTEM images of an Au@Pd NRB taken in the centre and at the edge, respectively (scale bars, 2 nm). (**n**) HAADF-STEM image (scale bar, 50 nm) and (**o**,**p**) the corresponding STEM-EDS elemental mappings of a typical Au@Pd NRB.

Significantly, the as-prepared 4H Au NRBs can serve as the substrates for the epitaxial growth of other noble metals with the similar 4H structure stabilization. For instance, Au@Ag NRBs were synthesized by reduction of AgNO_3_ with oleylamine in the presence of Au NRBs (see Methods for details). The ribbon shape is well preserved after the deposition of Ag on the surface of Au NRBs (Fig. 4a,b). The chemical composition of Au@Ag NRBs is examined using STEM-EDS, giving an average Au/Ag atomic ratio of 1.0/1.7 (Supplementary Fig. 13). High-angle annular dark-field-STEM (HAADF-STEM) images of a typical Au@Ag NRB and the corresponding STEM-EDS elemental maps indicate the uniform distribution of Au and Ag (Fig. 4f–h), which is further confirmed using the STEM-EDS line scanning analysis (Supplementary Fig. 14). Interestingly, the SAED pattern of a typical Au@Ag NRB shows the [110]_4H_ zone pattern together with streaks along the [001]_4H_ direction, suggesting the coexistence of 4H and *fcc* structures with stacking faults and twins present along the [001]_4H_/[111]_f_ direction (Fig. 4c). This indicates that the deposition of Ag on the surface of Au NRB led to the transformation of the original 4H Au to 4H/*fcc* polytypic structure. Such a structure was also confirmed using the selected-spot dark-field TEM analysis (Supplementary Fig. 15). HRTEM images of a typical Au@Ag NRB further prove the intergrowth of 4H and *fcc* phases (Fig. 4d,e), where the lattice fringes coherently extend from the centre to the edge of the Au@Ag NRB, indicating the epitaxial relationship between the Au core and the Ag shell (Fig. 4e). Moreover, most importantly, the 4H phase of Ag is stabilized through this epitaxial growth process. Similarly, polytypic 4H Pd and Pt can be stabilized via the same epitaxial growth process (Fig. 4i–p and Supplementary Figs 16–20). To the best of our knowledge, this is the first demonstration of 4H Pd and Pt nanostructure formation (Fig. 4k–m and Supplementary Fig. 18c–e).

## Discussion

In summary, a novel metastable phase of Au, that is, 4H Au, has been successfully prepared in high yield via a colloidal synthesis method. The ligand-induced phase transformation of Au NRBs from 4H to *fcc* has been observed under ambient conditions. The simulated and observed EELS spectra on a single Au NRB demonstrate that the optical response of 4H Au is quite different to that of *fcc* Au. With the epitaxial growth of other noble metals (for example, Ag, Pd and Pt) on the Au NRBs, 4H metastable phase of these noble metals can be synthesized. We believe that this series of novel noble metal nanostructures represent an important fundamental progress in chemistry and material science, and our synthesis will open up new opportunities for the controlled synthesis of functional nanomaterials that might have promising plasmonic and catalytic applications.

## Methods

### Materials

Gold (III) chloride hydrate (HAuCl_4_·aq, ∼50% Au basis), silver nitrate (AgNO_3_, ACS reagent, ≥99.0%), chloroplatinic acid hexahydrate (H_2_PtCl_6_·6H_2_O, ACS reagent, ≥37.5% Pt basis), palladium chloride (PdCl_2_, ≥99.9%), oleylamine (70%, technical grade), 1,2-dichloropropane (99%), 1-dodecanethiol (≥98%), 1-propanethiol (99%), 1,6-hexanedithiol (>97%), hexane (technical grade), chloroform (≥99.5%) and the other chemicals used in the experiments without special mention were all purchased from Sigma-Aldrich. Concentrated hydrochloric acid (HCl, 37%) and ethanol (99.9%, absolute) were purchased from Merck. Sodium borohydride (NaBH_4_, ≥99.0%) was purchased from Fluka. All the reagents were used as received without further purification.

### Synthesis of 4H Au NRBs

In a typical experiment, HAuCl_4_ (4.08 mg) and oleylamine (220 μl) were dissolved in hexane (3.54 ml) and 1,2-dichloropropane (250 μl). The mixture in a closed glass vial was then heated in a water bath at 58 °C for 16 h. After that, the resulting product was collected using centrifugation (5,000 r.p.m., 1 min), washed at least three times with hexane and then re-dispersed into hexane (4 ml). The yield of 4H Au NRBs is estimated to be ∼60% in the final products.

### Ligand exchange on 4H Au NRBs

Typically, a small amount of 4H Au NRB solution (300 μl, in hexane) was centrifuged (5,000 r.p.m., 1 min) and re-dispersed into chloroform (300 μl). Then, a fresh 1-dodecanethiol solution (0.8 M, in chloroform) was added and vortexed for 5 min under ambient conditions. After that, the solution was centrifuged (6,000 r.p.m., 1 min), washed twice with hexane and then re-dispersed in hexane before further characterization. Note that 1-dodecanethiol used here can be replaced by 1-propanethiol or 1,6-hexanedithiol while the other experimental conditions keep same.

### Synthesis of 4H/*fcc* Au@Ag core–shell NRBs

The 4H/*fcc* Au@Ag NRBs were synthesized through the epitaxial growth of Ag on 4H Au NRBs by using oleylamine as the reducing agent. Typically, 130 μl of oleylamine, 250 μl of AgNO_3_ solution (50 mM, ethanol) and 1.14 ml of hexane were successively added into 0.5 ml of the as-prepared 4H Au NRB solution (in hexane). The mixture in a closed glass vial was then heated in a water bath at 58 °C for 20 h. The resulting product was collected using centrifugation (5,000 r.p.m., 1 min), washed twice with hexane and re-dispersed in hexane (0.5 ml) before further characterization.

### Synthesis of 4H/*fcc* Au@Pd core–shell NRBs

H_2_PdCl_4_ stock solution (8 mM, in ethanol) was first prepared by mixing PdCl_2_ powder with concentrated HCl in ethanol. A small amount of 4H Au NRB solution (250 μl, in hexane) was centrifuged (5,000 r.p.m., 1 min) and re-dispersed into chloroform (250 μl). The 4H/*fcc* Au@Pd NRBs were synthesized through the epitaxial growth of Pd on 4H Au NRBs by using NaBH_4_ as the reducing agent. Typically, 250 μl of chloroform, 390 μl of ethanol, 50 μl of H_2_PdCl_4_ solution (8 mM, ethanol) and 60 μl of NaBH_4_ solution (20 mM, ethanol) were successively added into 250 μl of the as-prepared 4H Au NRB solution (in chloroform). The mixture was gently shaken and then kept undisturbed at room temperature for 1 h. The product was collected using centrifugation (5,000 r.p.m., 1 min), washed once with hexane and re-dispersed in hexane before further characterization.

### Synthesis of 4H/*fcc* Au@Pt core–shell NRBs

A small amount of 4H Au NRB solution (250 μl, in hexane) was centrifuged (5,000 r.p.m., 1 min) and re-dispersed into chloroform (250 μl). The 4H/*fcc* Au@Pt NRBs were synthesized through the epitaxial growth of Pt on 4H Au NRBs by using NaBH_4_ as the reducing agent. Typically, 250 μl of chloroform, 340 μl of ethanol, 100 μl of H_2_PtCl_6_ solution (8 mM, in ethanol) and 60 μl of NaBH_4_ solution (20 mM, in ethanol) were successively added into 250 μl of the as-prepared 4H Au NRB solution (in chloroform). The mixture was gently shaken and then kept undisturbed at room temperature for 1 h. The product was collected using centrifugation (5,000 r.p.m., 1 min), washed once with hexane and re-dispersed in hexane before further characterization.

### SPR study of single 4H Au NRB

The monochromated EELS measurement of a single Au NRB was conducted in the STEM mode using an FEI Titan TEM with Schottky electron source operated at 80 kV, using a convergence semiangle of 13 mrad and an EELS collection semiangle of 16 mrad. The diameter of used electron probe was ∼1–2 nm. The energy resolution was set to ∼0.1 eV (as full-width at half-maximum value), using a Wien-type monochromator. EELS spectroscopy and map were collected with a Gatan Tridiem ER EELS detector. EELS was acquired with a modified binned gain averaging acquisition routine^46^, to give improved signal-to-noise ratio for the relatively weak plasmon signal from few-nanometre-thick material. The background signal was taken from a bare amorphous SiN_*x*_ TEM support membrane, fitted to and subtracted from the experimental EELS plasmon spectra. EELS maps plotted the loss signal integrated over an energy window of 0.05 eV, centred around selected SPR peaks.

### DFT computational details

The dielectric function of 4H Au thin film is calculated by first principle calculations on the basis of the optical package of WIEN2k (refs 47, 48, 49). This package allows us to calculate the dipole matrix elements for transitions between intraband and interbands within the random phase approximation. The spin–orbit interactions are taken into account in all calculations. To increase the accuracy of the optical properties, we used a very high k-mesh of 40 × 40 × 1 for the computation.

### FEM simulation

The swift electron-driven excitation of plasmons in an individual 4H Au NRB was simulated using the FEM in the COMSOL Multiphysics RF module. The Au NRB was modelled as a perfect cuboid with the dimensions extracted from STEM image of the sample (length: 840 nm, width: 20 nm and thickness: 4 nm). The frequency-dependent dielectric function of Au was taken from a handbook^44^.

The electron beam was modelled in the framework of classical electrodynamics^50^,^51^ as an infinitely long broadband current *J*_z_(*ω*) propagating in the *z* direction with the radius of 1 nm corresponding to the kinetic energy of 80 keV. In our model, the beam was located at 105 nm (that is, position ‘I'), 210 nm (that is, position ‘II'), 315 nm (that is, position ‘III') and 420 nm (that is, position ‘IV') away from one end of the Au NRB. The surrounding medium was air/vacuum with *ɛ*=1. The 30-nm-thick Si_3_N_4_ substrate was ignored in the simulation set-up.

The electromagnetic field distribution was obtained by solving three-dimensional Maxwell's equations in the frequency domain. The power lost by the electron beam in the excitation of wave perturbations with frequency *ω* was calculated as the integral over the beam volume *V*,


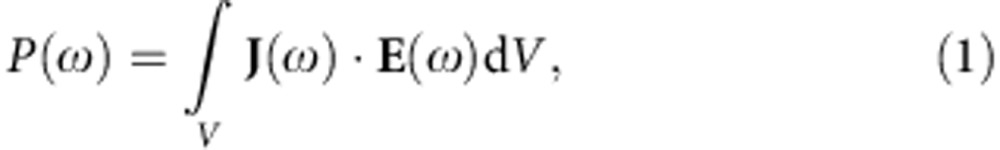


where **E**(*ω*) is the calculated distribution of electric field. Finally, the electron loss probability^51^,


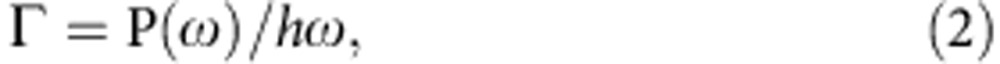


versus photon energy *hω* is compared with the EELS spectrum.

### Characterization

The TEM samples were prepared by directly dropping 5-μl solution of various samples on full carbon-coated copper grids (200 mesh) and then drying under ambient conditions. The aberration-corrected HRTEM images of 4H Au NRB were collected using the negative spherical aberration (*C*_S_) imaging (NCSI) technique on TEAM 0.5, which is an aberration-corrected microscope equipped with a high-brightness Schottky-type field emission gun and a Wien-filter monochromator. The accelerating voltage was 80 kV. The lens aberrations were measured and compensated before the image acquisition by evaluating the Zemlin tableau of an amorphous carbon area on the grid, which is close to the area of interest in the specimen. According to the measurements, the residual lens aberrations were listed below *C*_S_∼−13 μm, twofold astigmatism *A*1<2 nm, threefold astigmatism *A*2<40 nm, axis coma *B*2<30 nm. TEM and the other HRTEM images, SAED patterns and the HAADF-STEM-EDS data were taken on a JEOL JEM-2100F TEM operated at 200 kV. The AFM images were collected on an atomic force microscope (Dimension 3100, Veeco, USA). The X-ray photoelectron spectroscopy data were collected with a Theta Probe electron spectrometer (ESCA-Lab-200i-XL, Thermo Scientific). The X-ray diffraction patterns were obtained on an X-ray diffractometer (Shimadzu, XRD-6000) operated at 40 kV and 30 mA. The X-ray diffraction samples were prepared by dropping the sample solution on the glass substrate and then drying under ambient conditions.

## Additional information

**How to cite this article:** Fan, Z. *et al.* Stabilization of 4H hexagonal phase in gold nanoribbons. *Nat. Commun.* 6:7684 doi: 10.1038/ncomms8684 (2015).

## Supplementary Material

Supplementary Information Supplementary Figures 1-20 and Supplementary References

## References

[b1] Voiry D. *et al.* Enhanced catalytic activity in strained chemically exfoliated WS_2_ nanosheets for hydrogen evolution. Nat. Mater. 12, 850–855 (2013). 23832127 10.1038/nmat3700

[b2] Zhang J., Xu Q., Feng Z., Li M. & Li C. Importance of the relationship between surface phases and photocatalytic activity of TiO_2_. Angew. Chem. Int. Ed. 47, 1766–1769 (2008). 10.1002/anie.200704788 18213667

[b3] Thelander C., Caroff P., Plissard S., Dey A. W. & Dick K. A. Effects of crystal phase mixing on the electrical properties of InAs nanowires. Nano Lett. 11, 2424–2429 (2011). 21528899 10.1021/nl2008339

[b4] Chen C.-C., Herhold A. B., Johnson C. S. & Alivisatos A. P. Size dependence of structural metastability in semiconductor nanocrystals. Science 276, 398–401 (1997). 9103194 10.1126/science.276.5311.398

[b5] Caroff P. *et al.* Controlled polytypic and twin-plane superlattices in III-V nanowires. Nat. Nanotechol. 4, 50–55 (2009). 10.1038/nnano.2008.359 19119283

[b6] Park C. H., Cheong B.-H., Lee K.-H. & Chang K. J. Structural and electronic properties of cubic, 2H, 4H, and 6H SiC. Phys. Rev. B 49, 4485–4493 (1994). 10.1103/physrevb.49.4485 10011368

[b7] Navrotsky A., Mazeina L. & Majzlan J. Size-driven structural and thermodynamic complexity in iron oxides. Science 319, 1635–1638 (2008). 18356516 10.1126/science.1148614

[b8] Wang Z. *et al.* Morphology-tuned wurtzite-type ZnS nanobelts. Nat. Mater. 4, 922–927 (2005). 16284620 10.1038/nmat1522

[b9] Nam K. M. *et al.* Syntheses and characterization of wurtzite CoO, rocksalt CoO, and spinel Co_3_O_4_ nanocrystals: their interconversion and tuning of phase and morphology. Chem. Mater. 22, 4446–4454 (2010).

[b10] Kim Y.-H., Jun Y.-w., Jun B.-H., Lee S.-M. & Cheon J. Sterically induced shape and crystalline phase control of GaP nanocrystals. J. Am. Chem. Soc. 124, 13656–13657 (2002). 12431078 10.1021/ja027575b

[b11] Kim J., Lee Y. & Sun S. Structurally ordered FePt nanoparticles and their enhanced catalysis for oxygen reduction reaction. J. Am. Chem. Soc. 132, 4996–4997 (2010). 20297818 10.1021/ja1009629

[b12] Kusada K. *et al.* Discovery of face-centered-cubic ruthenium nanoparticles: facile size-controlled synthesis using the chemical reduction method. J. Am. Chem. Soc. 135, 5493–5496 (2013). 23557199 10.1021/ja311261s

[b13] Xia Y., Xiong Y., Lim B. & Skrabalak S. E. Shape-controlled synthesis of metal nanocrystals: simple chemistry meets complex physics? Angew. Chem. Int. Ed. 48, 60–103 (2009). 10.1002/anie.200802248 PMC2791829 19053095

[b14] Daniel M.-C. & Astruc D. Gold nanoparticles: assembly, supramolecular chemistry, quantum-size-related properties, and applications toward biology, catalysis, and nanotechnology. Chem. Rev. 104, 293–346 (2004). 14719978 10.1021/cr030698+

[b15] Dreaden E. C., Alkilany A. M., Huang X., Murphy C. J. & El-Sayed M. A. The golden age: gold nanoparticles for biomedicine. Chem. Soc. Rev. 41, 2740–2779 (2012). 22109657 10.1039/c1cs15237h PMC5876014

[b16] Turkevich J., Stevenson P. C. & Hillier J. A study of the nucleation and growth processes in the synthesis of colloidal gold. Discuss. Faraday Soc. 11, 55–75 (1951).

[b17] Masuda H. & Fukuda K. Ordered metal nanohole arrays made by a two-step replication of honeycomb structures of anodic alumina. Science 268, 1466–1468 (1995). 17843666 10.1126/science.268.5216.1466

[b18] Jana N. R., Gearheart L. & Murphy C. J. Wet chemical synthesis of high aspect ratio cylindrical gold nanorods. J. Phys. Chem. B 105, 4065–4067 (2001).

[b19] Nikoobakht B. & El-Sayed M. A. Preparation and growth mechanism of gold nanorods (NRs) using seed-mediated growth method. Chem. Mater. 15, 1957–1962 (2003).

[b20] Millstone J. E. *et al.* Observation of a quadrupole plasmon mode for a colloidal solution of gold nanoprisms. J. Am. Chem. Soc. 127, 5312–5313 (2005). 15826156 10.1021/ja043245a

[b21] Shankar S. S. *et al.* Biological synthesis of triangular gold nanoprisms. Nat. Mater. 3, 482–488 (2004). 15208703 10.1038/nmat1152

[b22] Lu X., Yavuz M. S., Tuan H.-Y., Korgel B. A. & Xia Y. Ultrathin gold nanowires can be obtained by reducing polymeric strands of oleylamine−AuCl complexes formed via aurophilic interaction. J. Am. Chem. Soc. 130, 8900–8901 (2008). 18540574 10.1021/ja803343m

[b23] Wang C., Hu Y., Lieber C. M. & Sun S. Ultrathin Au nanowires and their transport properties. J. Am. Chem. Soc. 130, 8902–8903 (2008). 18540579 10.1021/ja803408f

[b24] Huo Z., Tsung C.-k., Huang W., Zhang X. & Yang P. Sub-two nanometer single crystal Au nanowires. Nano Lett. 8, 2041–2044 (2008). 18537294 10.1021/nl8013549

[b25] Zhang J. *et al.* Sonochemical formation of single-crystalline gold nanobelts. Angew. Chem. Int. Ed. 118, 1134–1137 (2006). 10.1002/anie.200503762 16389606

[b26] Kim F., Connor S., Song H., Kuykendall T. & Yang P. Platonic gold nanocrystals. Angew. Chem. Int. Ed. 116, 3759–3763 (2004). 10.1002/anie.200454216 15248270

[b27] Sun Y. & Xia Y. Shape-controlled synthesis of gold and silver nanoparticles. Science 298, 2176–2179 (2002). 12481134 10.1126/science.1077229

[b28] Taneja P., Banerjee R., Ayyub P. & Dey G. K. Observation of a hexagonal (4H) phase in nanocrystalline silver. Phys. Rev. B 64, 033405 (2001).

[b29] Liu X., Luo J. & Zhu J. Size effect on the crystal structure of silver nanowires. Nano Lett. 6, 408–412 (2006). 16522032 10.1021/nl052219n

[b30] Chakraborty I. *et al.* Novel hexagonal polytypes of silver: growth, characterization and first-principles calculations. J. Phys. Condens. Matter 23, 325401 (2011). 21785182 10.1088/0953-8984/23/32/325401

[b31] Chakraborty I., Shirodkar N. S., Gohil S., Waghmare V. U. & Ayyub P. The nature of the structural phase transition from the hexagonal (4H) phase to the cubic (3C) phase of silver. J. Phys. Condens. Matter 26, 115405 (2014). 24589655 10.1088/0953-8984/26/11/115405

[b32] Chakraborty I., Shirodkar N. S., Gohil S., Waghmare V. U. & Ayyub P. A stable, quasi-2D modification of silver: optical, electronic, vibrational and mechanical properties, and first principles calculations. J. Phys. Condens. Matter 26, 025402 (2014). 24305516 10.1088/0953-8984/26/2/025402

[b33] Huang X. *et al.* Synthesis of hexagonal close-packed gold nanostructures. Nat. Commun. 2, 292 (2011). 21522136 10.1038/ncomms1291

[b34] Loubat A. *et al.* Growth and self-assembly of ultrathin Au nanowires into expanded hexagonal superlattice studied by *in situ* SAXS. Langmuir 30, 4005–4012 (2014). 24665883 10.1021/la500549z

[b35] Huang X. *et al.* Graphene oxide-templated synthesis of ultrathin or tadpole-shaped Au nanowires with alternating *hcp* and *fcc* domains. Adv. Mater. 24, 979–983 (2012). 22252895 10.1002/adma.201104153

[b36] Sebastian M. T. X-ray diffraction effects from 2H crystals undergoing transformation to the 4H structure by the shear mechanism. Cryst. Res. Technol. 22, 441–447 (1987).

[b37] Palosz B., Steurer W. & Schulz H. The structure of PbI_2_ polytypes 2H and 4H: a study of the 2H-4H transition. J. Phys. Condens. Matter 2, 5285 (1990).

[b38] Bauer R., Jägle E. A., Baumann W. & Mittemeijer E. J. Kinetics of the allotropic hcp–fcc phase transformation in cobalt. Phil. Mag. 91, 437–457 (2010).

[b39] Limpijumnong S. & Lambrecht W. R. L. Theoretical study of the relative stability of wurtzite and rocksalt phases in MgO and GaN. Phys. Rev. B 63, 104103 (2001).

[b40] Titmuss S., Wander A. & King D. A Reconstruction of clean and adsorbate-covered metal surfaces. Chem. Rev. 96, 1291–1306 (1996). 11848790 10.1021/cr950214c

[b41] Harris P. J. F. Sulphur-induced faceting of platinum catalyst particles. Nature 323, 792–794 (1986).

[b42] Vericat C., Vela M. E., Benitez G., Carro P. & Salvarezza R. C. Self-assembled monolayers of thiols and dithiols on gold: new challenges for a well-known system. Chem. Soc. Rev. 39, 1805–1834 (2010). 20419220 10.1039/b907301a

[b43] Bosman M. *et al.* Encapsulated annealing: enhancing the plasmon quality factor in lithographically-defined nanostructures. Sci. Rep. 4, 5537 (2014). 24986023 10.1038/srep05537 PMC4078311

[b44] Palik E. D. Handbook of Optical Constants of Solids Academic Press (1997).

[b45] Hövel M., Gompf B. & Dressel M. Dielectric properties of ultrathin metal films around the percolation threshold. Phys. Rev. B 81, 035402 (2010).

[b46] Bosman M. & Keast V. J. Optimizing EELS acquisition. Ultramicroscopy 108, 837–846 (2008). 18375066 10.1016/j.ultramic.2008.02.003

[b47] Blaha P., Schwarz K., Madsen G. K., Kvasnicka H. D. & Luitz J. WIEN2k, an Augmented Plane Wave Plus Local Orbitals Program for Calculating Crystal Properties Vienna University of Technology (2009).

[b48] Ambrosch-Draxl C. & Sofo J. O. Linear optical properties of solids within the full-potential linearized augmented planewave method. Comp. Phys. Commun. 175, 1–14 (2006).

[b49] Abt R., Ambrosch-Draxl C. & Knoll P. Optical response of high temperature superconductors by full potential LAPW band structure calculations. Phys. B 194–196, 1451–1452 (1994).

[b50] Jackson J. D. Classical Electrodynamics J. Wiley & Sons (1999).

[b51] García de Abajo F. J. Optical excitations in electron microscopy. Rev. Mod. Phys. 82, 209–275 (2010).

