# Supplementary material for: Stabilization of 4H hexagonal phase in gold nanoribbons
Source: Nat Commun. 2015 Jul 28;6:7684. doi: 10.1038/ncomms8684 (PMC4525209; doi:10.1038/ncomms8684)
Supplement: Supplementary Information — Supplementary Figures 1-20 and Supplementary References [file ncomms8684-s1.pdf]

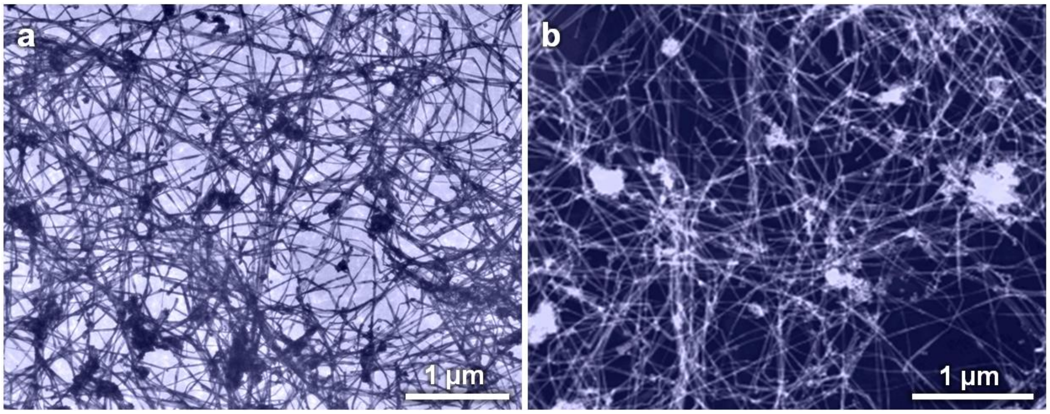

Supplementary Figure 1. Low-magnification electron microscope images of 4H Au nanoribbons (NRBs). Typical (**a**) TEM and (**b**) HAADF-STEM images of the as-prepared 4H Au NRBs. The dark/bright small dots and their assemblies are the by-products, i.e. *fcc* Au nanoparticles. The low-magnification images clearly show the micro-scale length of as-prepared Au NRBs.

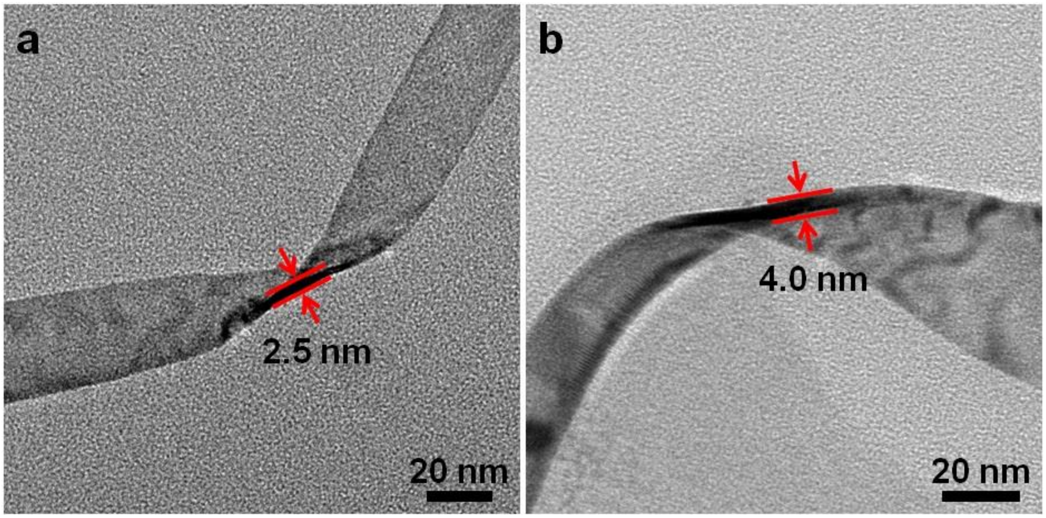

Supplementary Figure 2. Thickness measurement of 4H Au NRBs by TEM. Typical TEM images of two folded 4H Au NRBs, indicating a thickness of (**a**) 2.5 nm and (**b**) 4.0 nm, respectively. The as-prepared Au NRBs are very flexible due to their ultra-small thickness. Therefore, they can fold.

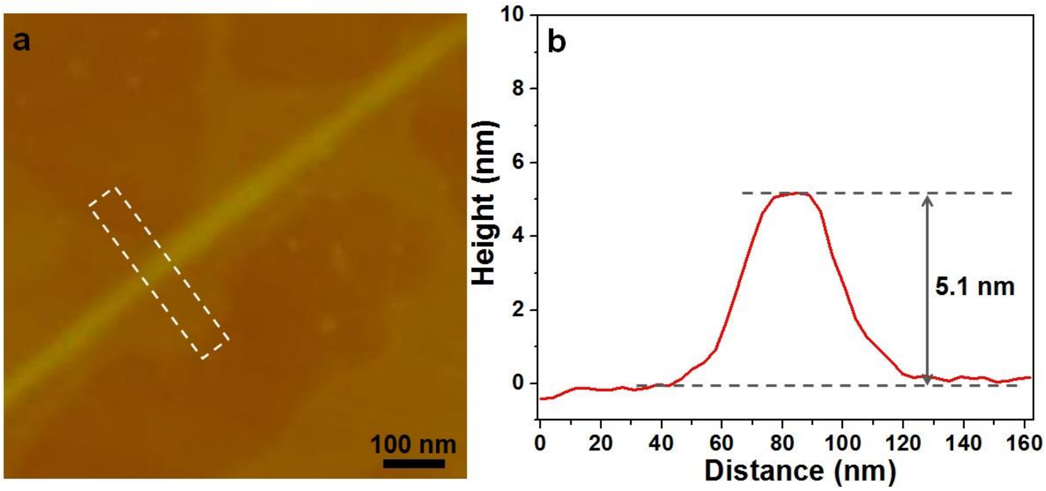

Supplementary Figure 3. Thickness measurement of the 4H Au NRB by AFM. (**a**) AFM image and (**b**) the corresponding height profile of a typical 4H Au NRB. The 4H Au NRBs were deposited on a Si/SiO_2 wafer and naturally dried. The AFM image was acquired with a scanning line of 512 and scanning rate of 1 Hz on a Dimension 3100 AFM (Veeco, USA), with a Si tip (spring constant: 42 N/m; resonance frequency: 320 kHz) in the tapping mode under ambient conditions. The measured thickness of Au NRB is about 5.1 nm, which is larger than those measured in Supplementary Fig. 2, due to the adsorption of oleylamine molecules (~1.6 nm in length) on the surface of Au NRBs[1,2].

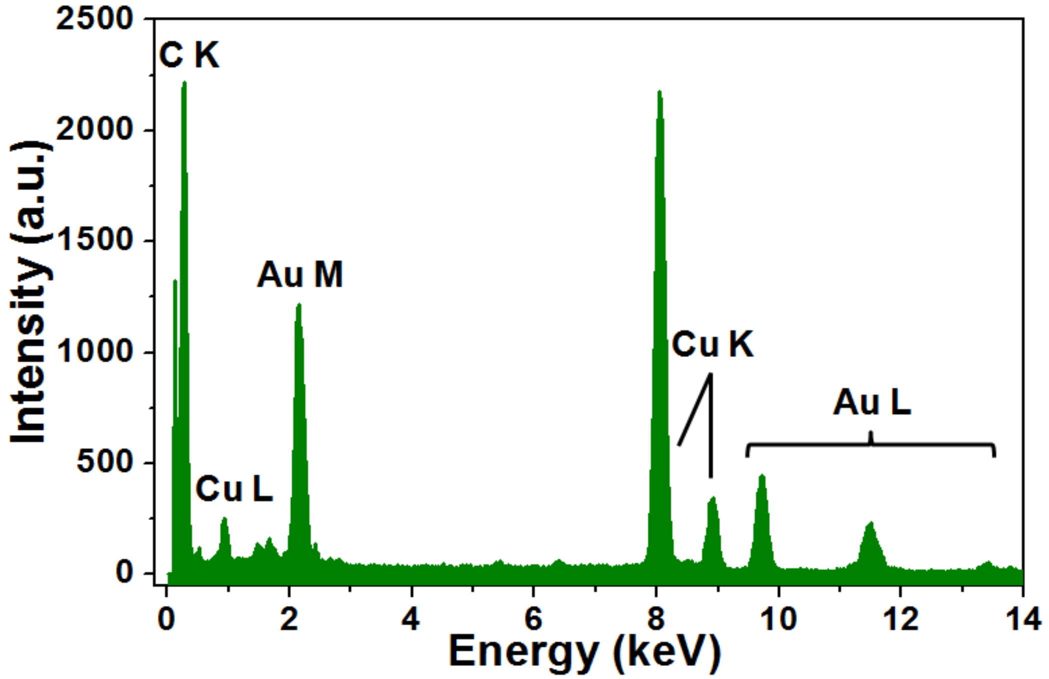

Supplementary Figure 4. Chemical composition analysis of 4H Au NRBs by STEM-EDS. A STEM-EDS spectrum obtained by combination of several spectra collected on individual Au nanoribbons.

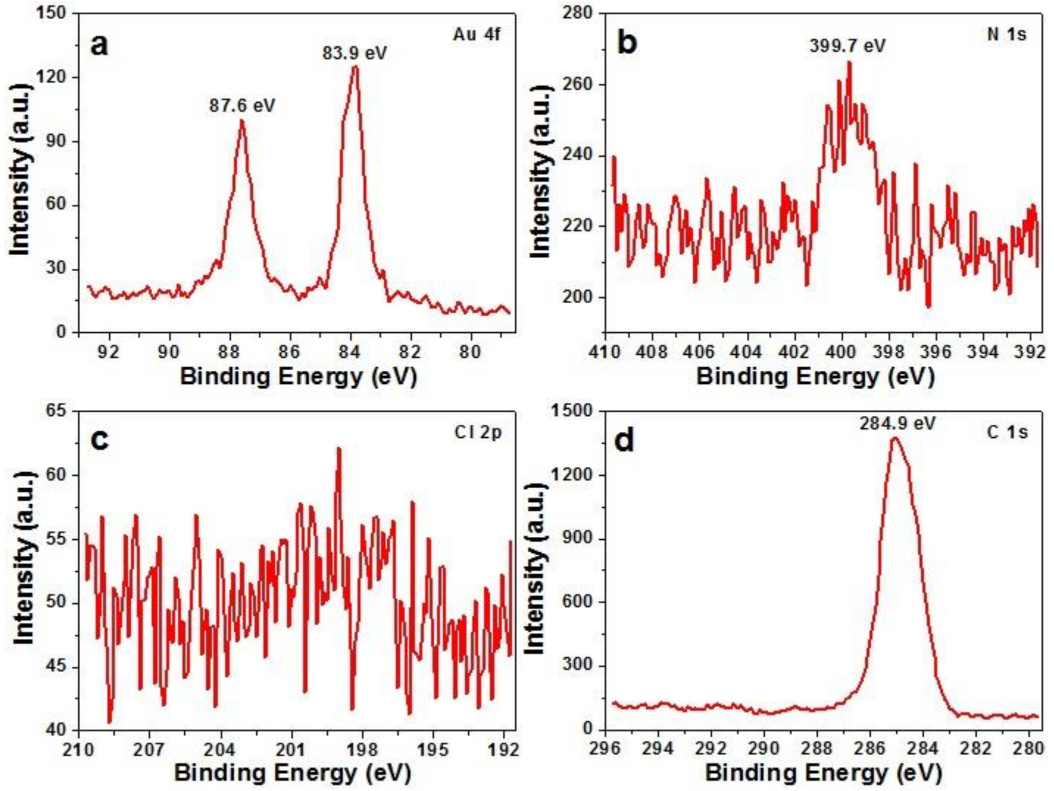

Supplementary Figure 5. Chemical composition analysis of 4H Au NRBs by XPS. XPS spectra of 4H Au NRBs showing the core level peaks of (**a**) Au4f, (**b**) N1s, (**c**) Cl2p, and (**d**) C1s. The N1s and C1s signals arise from the oleylamine molecules capped on the Au NRBs[2]. The presence of N1s peak indicates that the obtained Au NRBs are protected by oleylamine molecules.

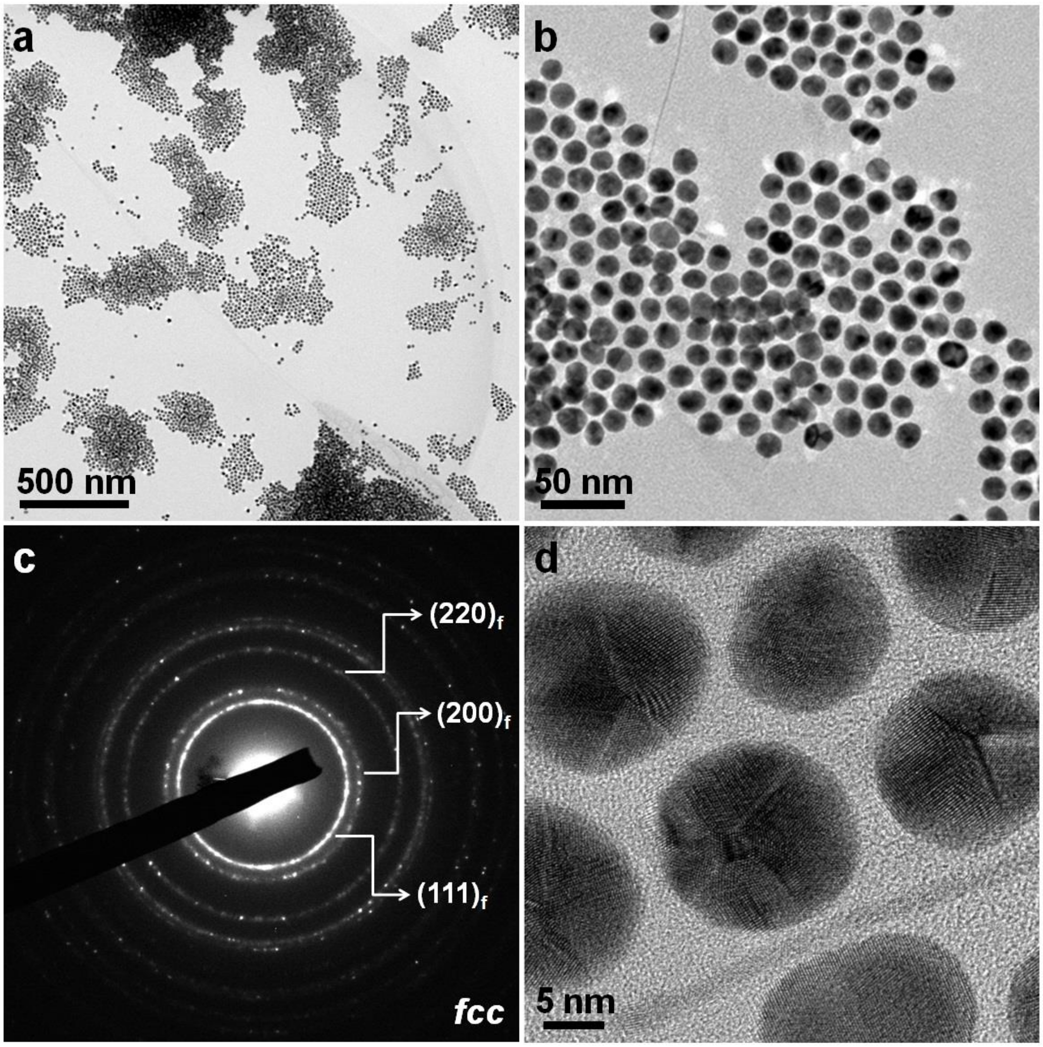

Supplementary Figure 6. The Au nanostructures obtained in the absence of 1,2-dichloropropane. (**a**) Low-magnification and (**b**) high-magnification TEM images of the obtained Au nanoparticles coexisting with very small amount of Au nanowires synthesized in the absence of 1,2-dichloroparopane. (**c**) The corresponding SAED pattern and (**d**) HRTEM image confirm that the obtained Au nanoparticle is multi-twinned *fcc* structure. Without the addition of 1,2-dichloropropane, no 4H Au NRBs are formed.

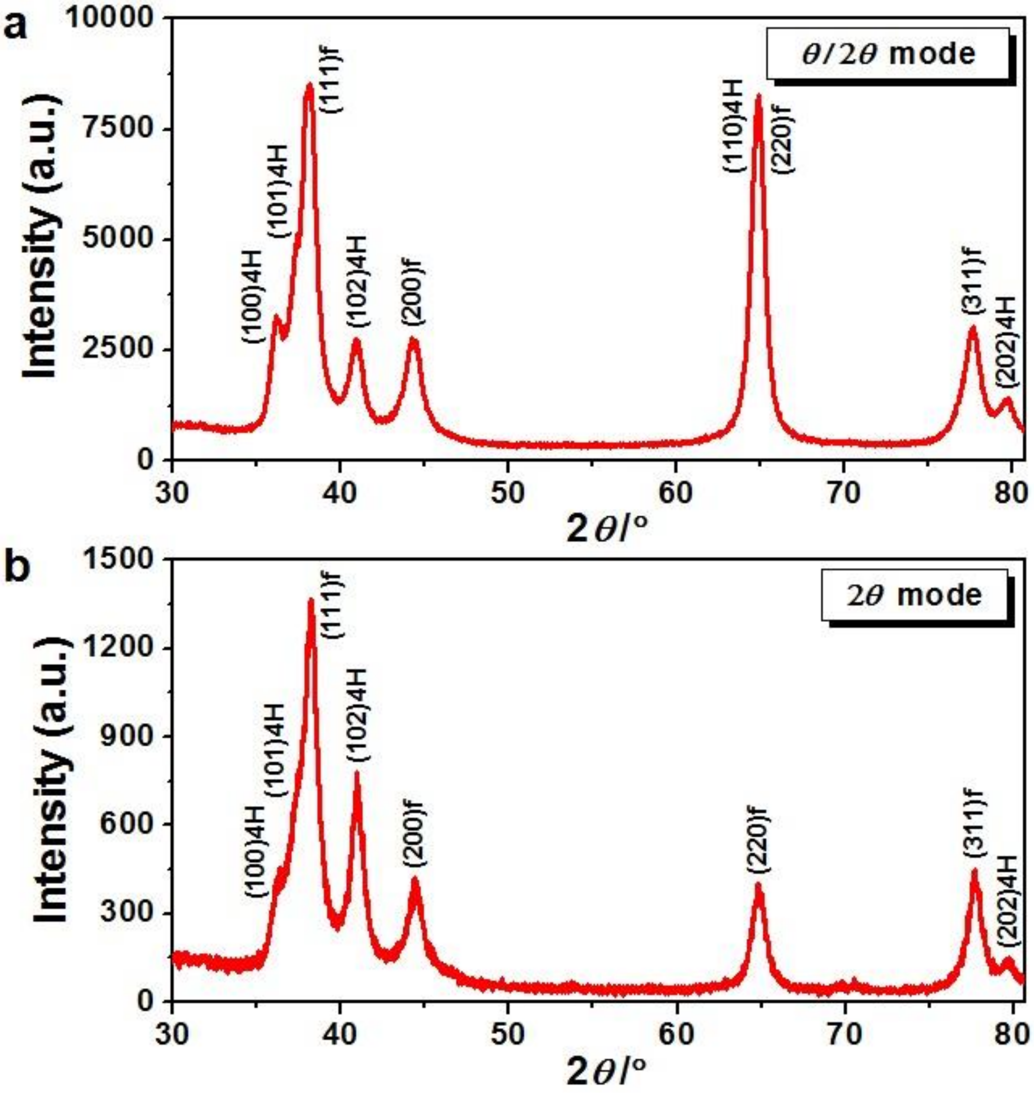

Supplementary Figure 7. XRD analysis of the crystal structure of 4H Au NRBs. (**a**) XRD pattern of Au NRBs collected in the $\theta/2\theta$ mode. In this mode, $(110)_{4H}$ and $(220)_{f}$ peaks are overlapped due to their equal lattice spacing. (**b**) XRD pattern of Au NRBs collected in the 2θ mode. In this mode, the intensity of $(110)_{4H}$ peak is neglectable as the Au NRBs tend to lay down on the substrate. As a result, the relative intensity of the peak at 64.8° obtained in the 2θ mode is much weaker compared to that in the $\theta/2\theta$ mode[1]. The presence of four peaks at 36.2°, 37.4°, 40.9° and 79.7°, which are attributed to the $(100)_{4H}$, $(101)_{4H}$, $(102)_{4H}$ and $(202)_{4H}$ planes of 4H Au, confirms the 4H hexagonal structure of the obtained Au NRBs.

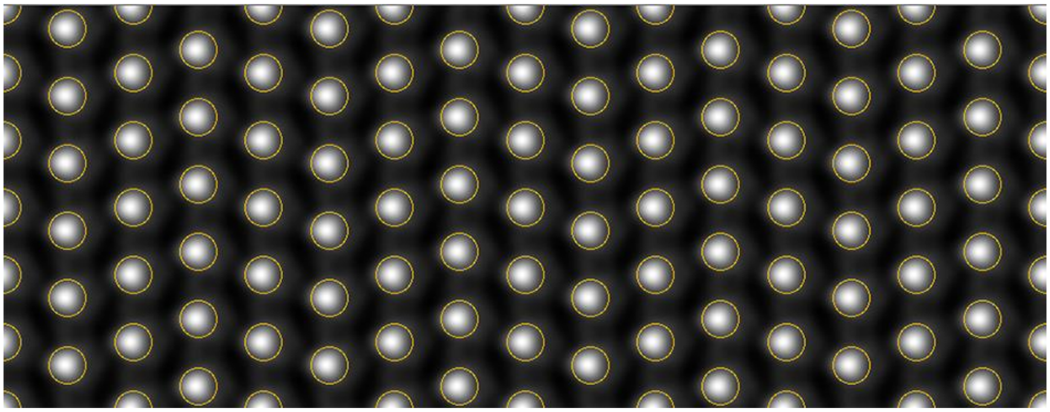

Supplementary Figure 8. Simulated HRTEM image of the 4H Au NRB. HRTEM image simulation was carried out using the multislice method as implemented in the MacTempas software. The simulated image, showing the best fit of the experimental images in Fig. 1d,e was obtained at defocus value $C1 = +10$ nm, two-fold astigmatism $A1 = 1$ nm, three-fold astigmatism $A2 = 30$ nm, coma $B2 = 10$ nm, sample thickness $t = 3$ nm, crystal tilt of 2.0 mrad toward the [001] direction, and vibration of 0.03, 0.03 nm. The simulated image was shown overlapped with an atom projection. There is a one-to-one correspondence between the intensity maxima and the projected Au atom positions. The good match between the simulated and experimental HRTEM images further confirms the 4H hexagonal structure of the obtained Au NRBs.

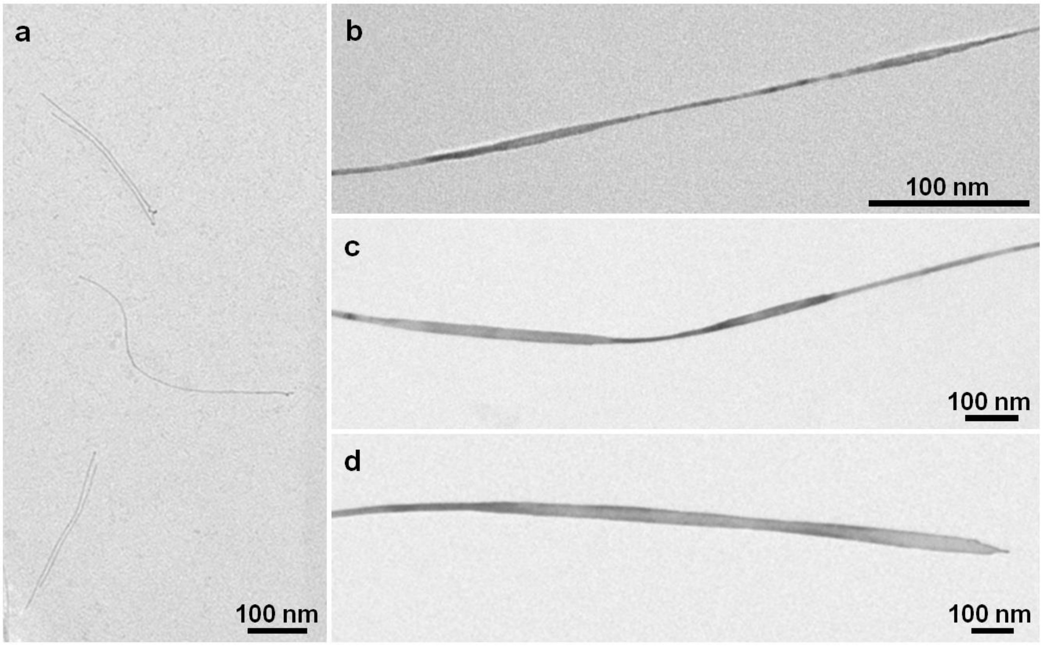

Supplementary Figure 9. TEM images illustrating the shape evolution of the Au NRB. (**a**) At the reaction time of 4 h, ultrathin Au nanowires (1.4–2.0 nm in diameter) were formed. The Au nanowires grew over time to form Au NRBs with width of (**b**) 2.8–5.2 nm at 8 h, (**c**) 8.0–20.0 nm at 12 h, and (**d**) 15.0–61.0 nm at 16 h. In addition to the increase in width and thickness, the length of Au nanoribbons also gradually increased with the elongation of reaction time.

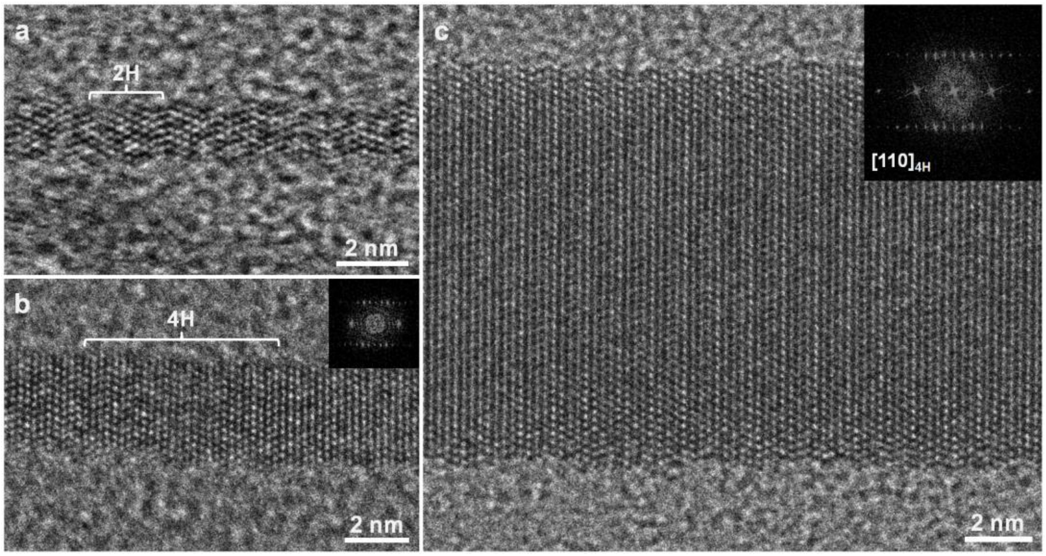

Supplementary Figure 10. HRTEM images illustrating the structure evolution of the Au NRB. (**a**) At the reaction time of 4 h, 2H structure and stacking faults were observed in the obtained ultrathin Au nanowires. Note that despite the special care taken during the capture of HRTEM images of ultrathin Au nanowires, we still observed the e-beam induced transformation of some 2H structures into disordered stacking during TEM characterization[3]. Besides, the e-beam induced transformation from 2H to disordered stacking has also been observed in the ultrathin Au square sheets[2]. (**b**) 4H structure appeared at the reaction time of 8 h. Inset: the corresponding FFT pattern of the marked 4H domain in **b**. (**c**) Au NRBs with the 4H structure were obtained at the reaction time of 12 h. Inset: the corresponding FFT pattern of the HRTEM image shown in **c**. The crystal structure of Au NRBs was obtained via the phase transition between different polytypes of Au, i.e. from the 2H to 4H polytypes.

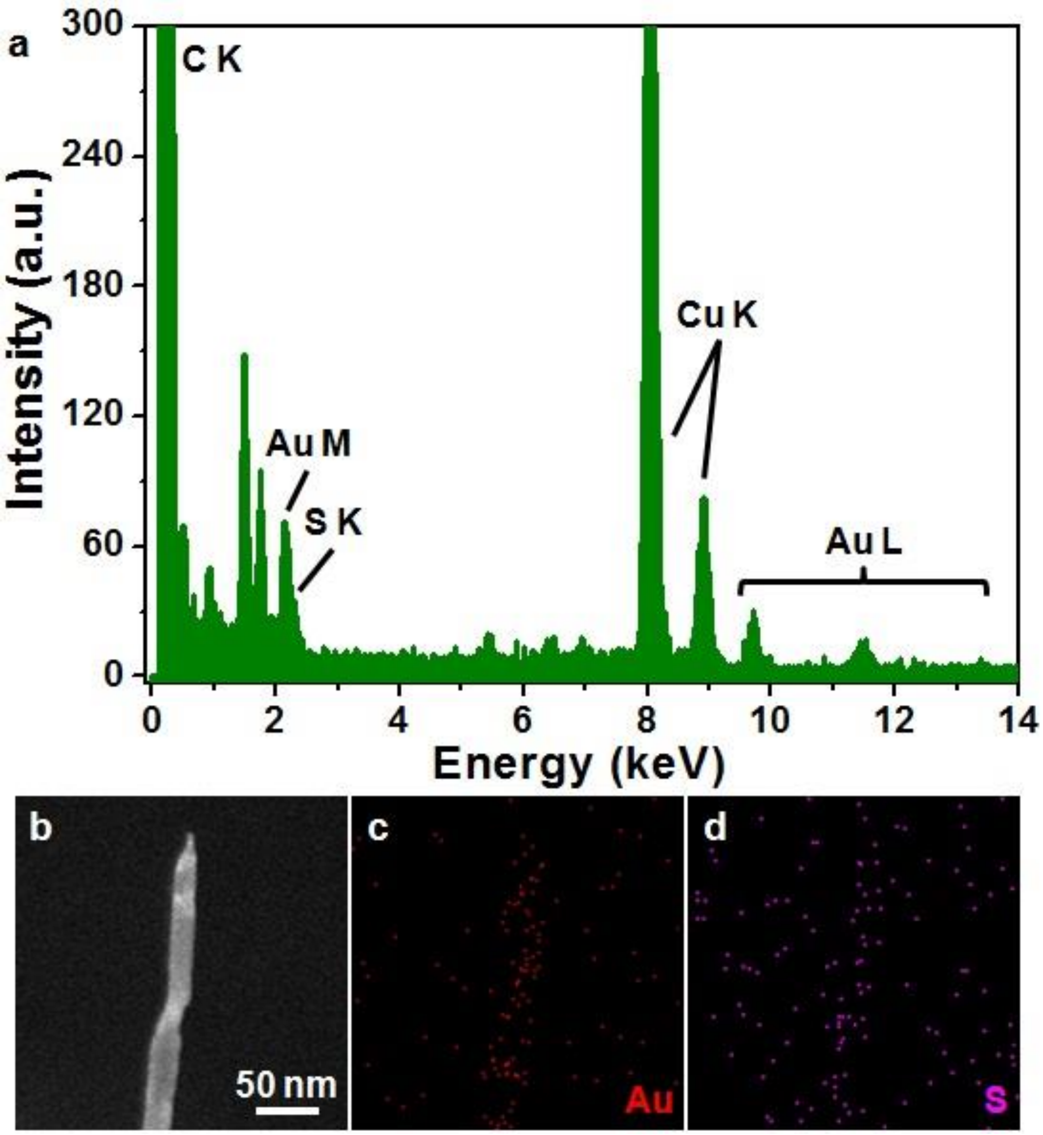

Supplementary Figure 11. Chemical composition analysis of Au NRBs after the ligand exchange. (**a**) A typical STEM-EDS spectrum of Au NRBs after the ligand exchange. The average Au/S atomic ratio is 1.0/0.6. (**b**) HAADF-STEM image and (**c**,**d**) the corresponding STEM-EDS elemental maps of a typical Au NRB after the ligand exchange. The presence of S indicates the occurrence of ligand exchange from oleylamine to thiol on the surface of Au NRBs.

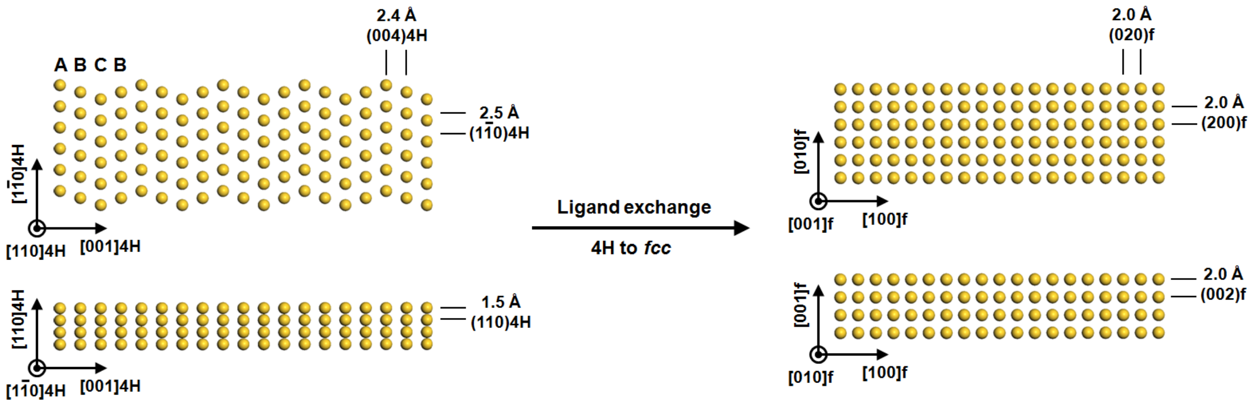

Supplementary Figure 12. Schematic illustration of the ligand exchange-induced phase transition of Au NRBs from 4H to *fcc* structures. This transition might arise from the flattening of the $(1\bar{1}0)_{4H}$ planes, which is quite different from the commonly observed phase transformations in the close-packed polytypes of metals/alloys[4-6]. The similar process has previously been observed in the phase transformation of GaN and CdSe nanostructures[7-9].

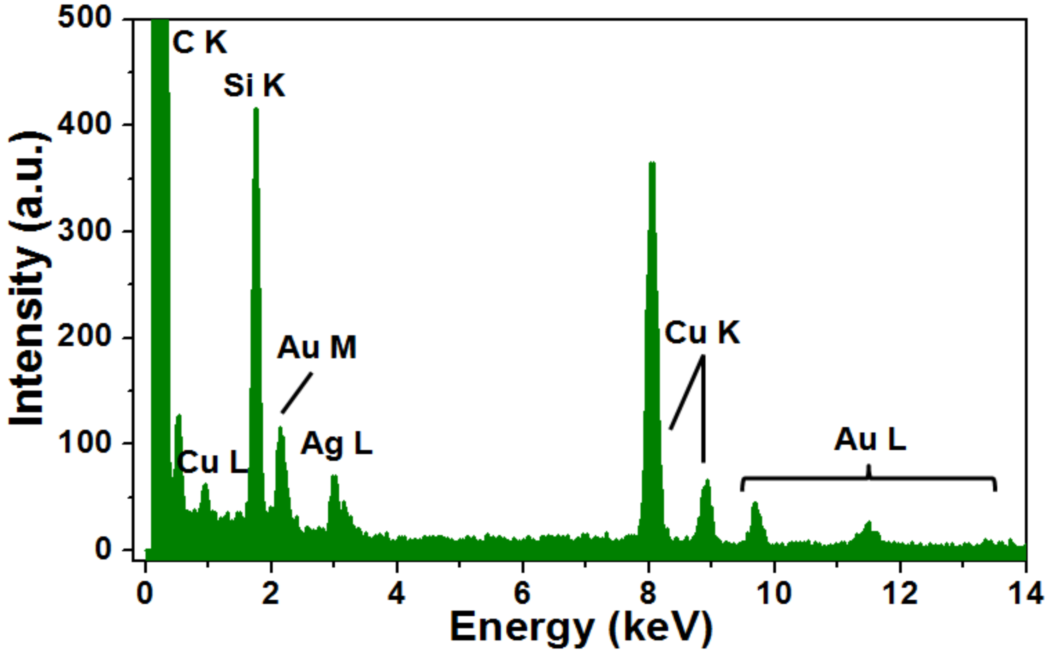

Supplementary Figure 13. Chemical composition analysis of the obtained 4H/*fcc* Au@Ag NRBs. A typical STEM-EDS spectrum of 4H/*fcc* Au@Ag NRBs with an average atomic ratio of 1.0/1.7 (Au/Ag), indicating the successful coating of Ag on the surface of Au NRBs.

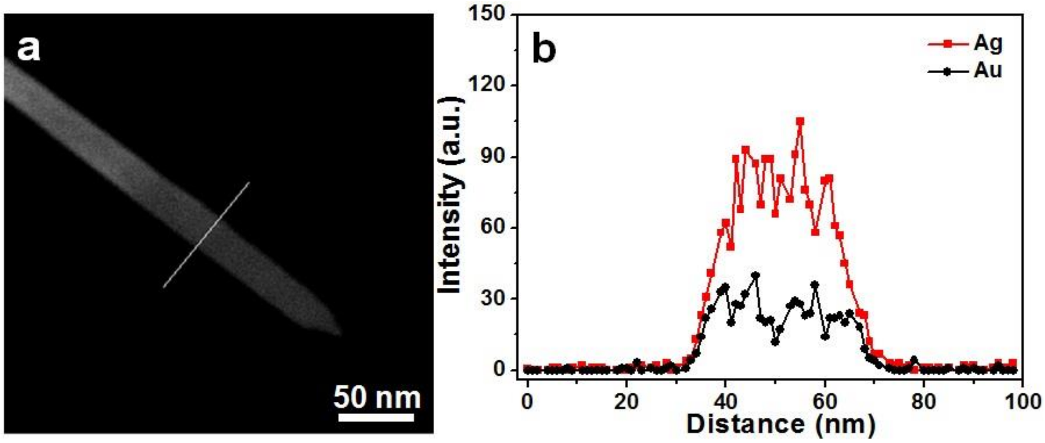

Supplementary Figure 14. STEM-EDS line scanning investigation of the obtained 4H/*fcc* Au@Ag NRBs. (**a**) HAADF-STEM image and (**b**) the corresponding STEM-EDS line scanning profile of a typical 4H/*fcc* Au@Ag NRB. The line scanning profile in **b** further confirms the uniform distribution of Au and Ag in the obtained Au@Ag NRBs.

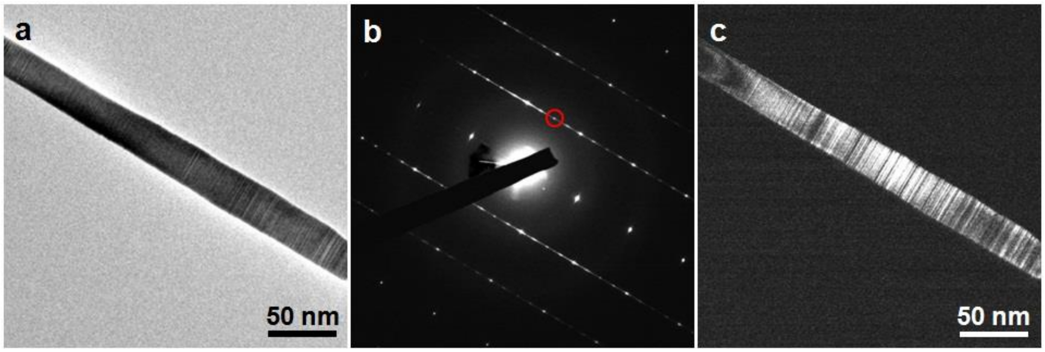

Supplementary Figure 15. Dark-field TEM analysis of the obtained 4H/*fcc* Au@Ag NRBs. (**a**) Bright-field TEM image and (**b**) the corresponding SAED pattern of a typical 4H/*fcc* Au@Ag NRB. (**c**) Dark-field TEM image taken with the $(1\,\bar{1}\,0)_{4H}$ reflection as marked in **b**. By comparison of **a** and **c**, the dark strips showing in **c** are attributed to the non-4H structures. The alternating distribution of 4H and *fcc* domains in **c** further confirms the polytypic 4H/*fcc* structure of the obtained Au@Ag NRBs.

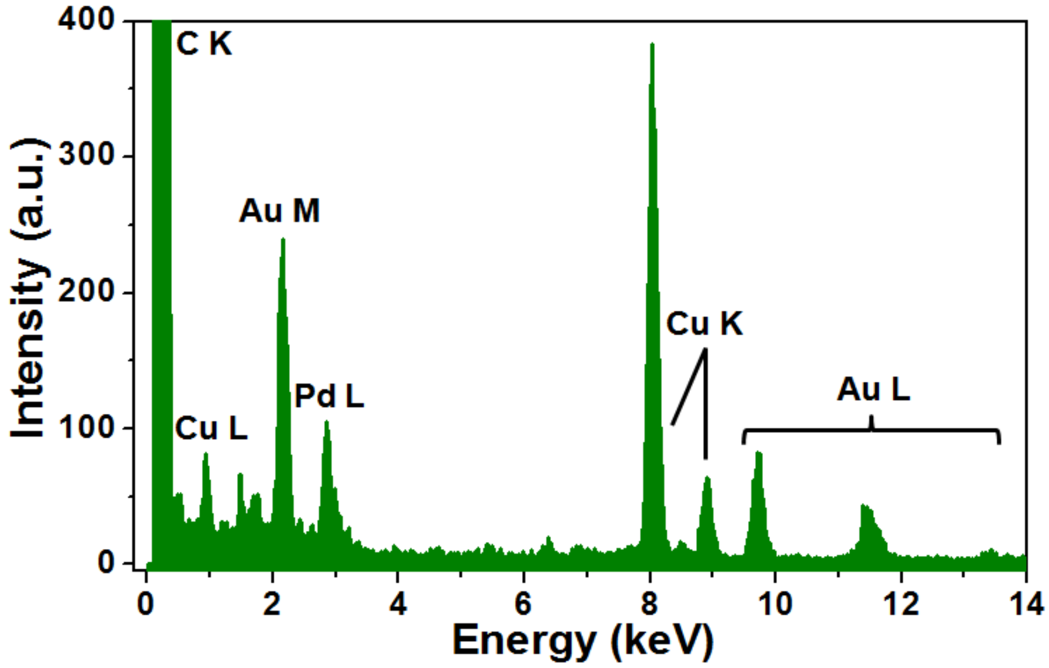

Supplementary Figure 16. Chemical composition analysis of the obtained 4H/*fcc* Au@Pd NRBs. A typical STEM-EDS spectrum of 4H/*fcc* Au@Pd NRBs with an average atomic ratio of 1.0/1.2 (Au/Pd), indicating the successful coating of Pd on the surface of Au NRBs.

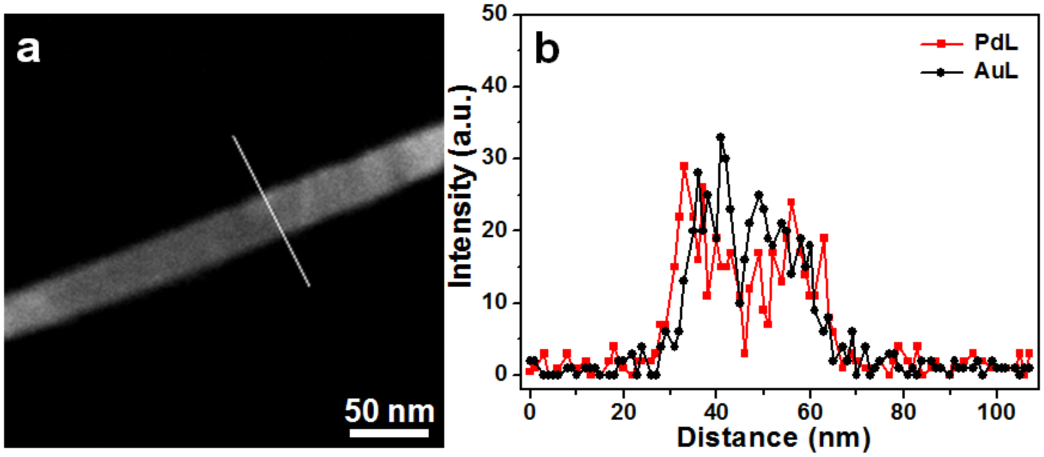

Supplementary Figure 17. STEM-EDS line scanning investigation of the obtained 4H/*fcc* Au@Pd NRBs. (**a**) HAADF-STEM image and (**b**) the corresponding STEM-EDS line scanning profile of a typical 4H/*fcc* Au@Pd NRB. The line scanning profile in **b** further confirms the uniform distribution of Au and Pd in the obtained Au@Pd NRBs.

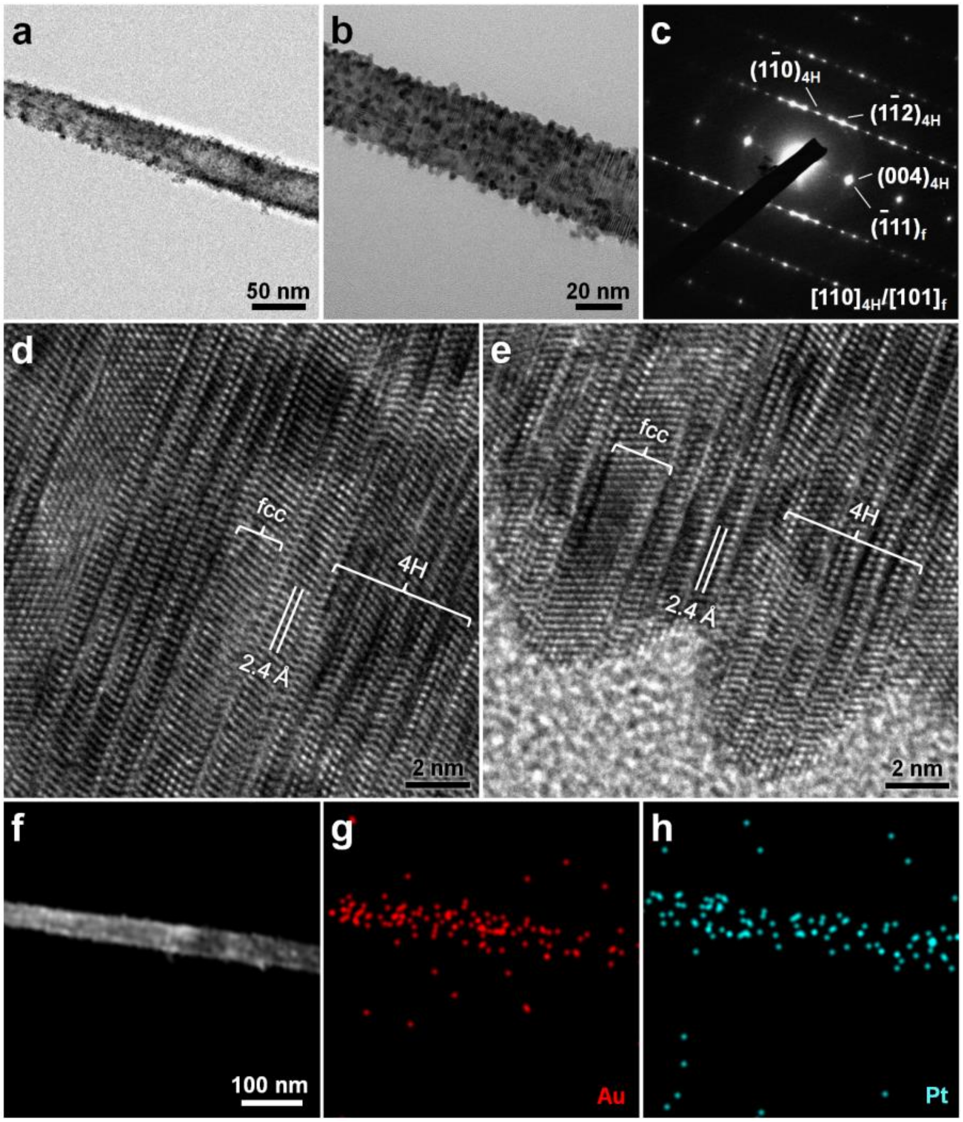

Supplementary Figure 18. TEM characterization of the obtained 4H/*fcc* Au@Pt NRBs. (**a**) Low- and (**b**) High-magnification TEM images, and (**c**) the corresponding SAED pattern of a typical 4H/*fcc* Au@Pt NRB. (**d**,**e**) HRTEM images of Au@Pt NRB taken in the center and at the edge, respectively. (**f**) HAADF-STEM image and (**g**,**h**) the corresponding STEM-EDS elemental map of a typical 4H/*fcc* Au@Pt NRB. The SAED pattern and HRTEM images confirm the 4H/*fcc* structure of the obtained Au@Pt NRBs and epitaxial growth of Pt on Au. The elemental mappings in **g** and **h** show the uniform distribution of Au and Pt in the obtained Au@Pt NRBs.

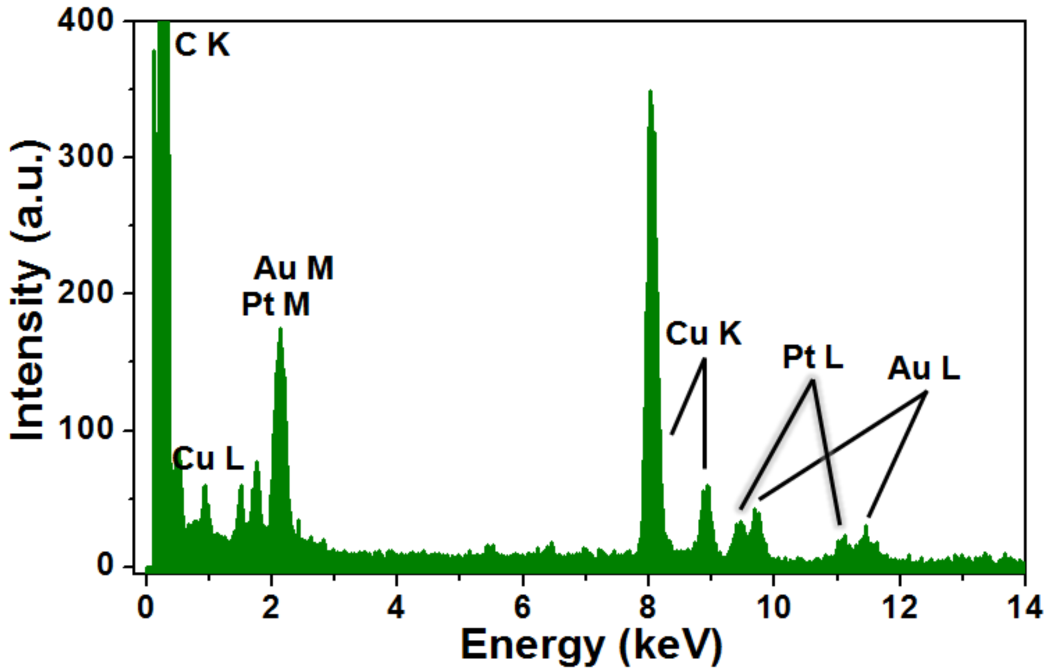

Supplementary Figure 19. Chemical composition analysis of the obtained 4H/*fcc* Au@Pt NRBs. A typical STEM-EDS spectrum of 4H/*fcc* Au@Pt NRBs with an average atomic ratio between Au and Pt of 1.0/0.9, indicating the successful coating of Pt on the surface of Au NRBs.

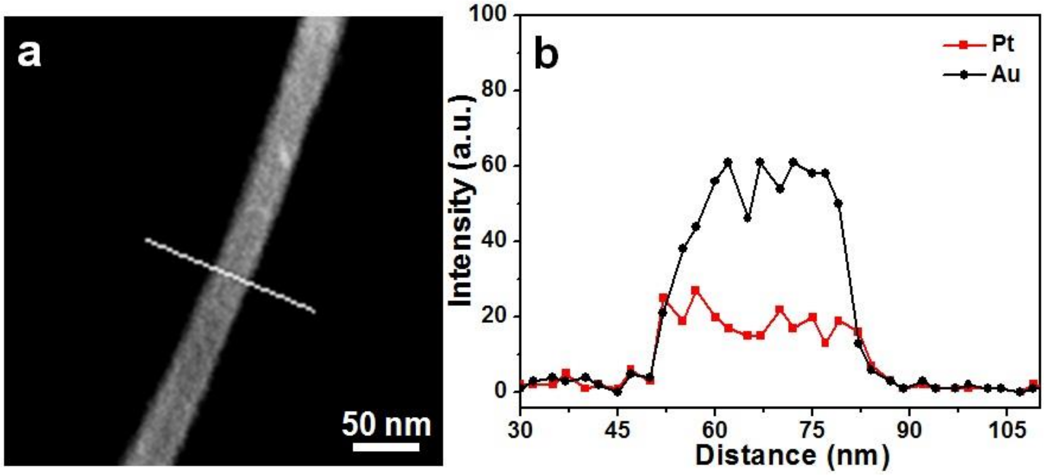

Supplementary Figure 20. STEM-EDS line scanning investigation of the obtained 4H/*fcc* Au@Pt NRBs. (**a**) HAADF-STEM image and (**b**) the corresponding STEM-EDS line scanning profile of a typical 4H/*fcc* Au@Pt NRB. The line scanning profile in **b** further confirms the uniform distribution of Au and Pt in the obtained Au@Pt NRBs.

Supplementary Reference

1. Huang, X. *et al.* Synthesis of gold square-like plates from ultrathin gold square sheets: the evolution of structure phase and shape. *Angew. Chem. Int. Ed.* **50**, 12245-12248 (2011).
2. Huang, X. *et al.* Synthesis of hexagonal close-packed gold nanostructures. *Nat. Commun.* **2**, 292 (2011).
3. Huang, X. *et al.* Graphene oxide-templated synthesis of ultrathin or tadpole-shaped Au nanowires with alternating *hcp* and *fcc* domains. *Adv. Mater.* **24**, 979-983 (2012).
4. Bauer, R., Jägle, E. A., Baumann, W. & Mittemeijer, E. J. Kinetics of the allotropic hcp–fcc phase transformation in cobalt. *Phil. Mag.* **91**, 437-457 (2010).
5. Tolédano, P., Krexner, G., Prem, M., Weber, H. P. & Dmitriev, V. P. Theory of the martensitic transformation in cobalt. *Phys. Rev. B* **64**, 144104 (2001).
6. Blaschko, O. *et al.* Coherent modulated structure during the Martensitic hcp-fcc phase transition in Co and in a CoNi alloy. *Phys. Rev. Lett.* **60**, 2800-2803 (1988).
7. Limpijumnong, S. & Lambrecht, W. R. L. Theoretical study of the relative stability of wurtzite and rocksalt phases in MgO and GaN. *Phys. Rev. B* **63**, 104103 (2001).
8. Grünwald, M., Rabani, E. & Dellago, C. Mechanisms of the wurtzite to rocksalt transformation in CdSe nanocrystals. *Phys. Rev. Lett.* **96**, 255701 (2006).
9. Chen, C.-C., Herhold, A. B., Johnson, C. S. & Alivisatos, A. P. Size dependence of structural metastability in semiconductor nanocrystals. *Science* **276**, 398-401 (1997).
